# Co-enzyme-free, enzymatic synthesis of aldehydes from renewable resources with a new and highly efficient alkene cleaving dioxygenase

**DOI:** 10.1039/d5gc01848j

**Published:** 2025-08-04

**Authors:** Astrid Schiefer, Lukas Schober, Thomas Rohr, Margit Winkler, Florian Rudroff

**Affiliations:** a Institute of Applied Synthetic Chemistry, TU Wien Getreidemarkt 9 1060 Vienna Austria florian.rudroff@tuwien.ac.at; b Institute of Molecular Biotechnology, Graz University of Technology, NAWI Graz Petersgasse 14 Graz Austria margit.winklertugraz.at; c Austrian Center of Industrial Biotechnology Krenngasse 37 Graz Austria

## Abstract

Alkene cleaving dioxygenases (ADO), which can oxidatively cleave C=C double bonds to the respective carbonyl compounds, may aid in waste stream utilization strategies by valorizing lignin-derived monomers. Here, we present 11 new ADOs and describe the characteristics of the most promising candidate *Map*ADO from *Moesziomyces aphidis*. *Map*ADO shows unprecedented reaction kinetics and a high yield without requiring co-enzymes or co-substrates other than O_2_. We highlight the efficiency of *Map*ADO by preparative scale reaction in a whole cell approach from 50 mM isoeugenol to vanillin isolated in 85% yield. Furthermore, we demonstrate that *Map*ADO can be employed in a cascade reaction with a eugenol oxidase (*Sc*EUGO), enabling the use of eugenol as starting material. Moreover, we show that *Map*ADO can be used to convert ferulic acid – a major component of lignin – to vanillin *via* another cascade reaction employing a phenolic acid decarboxylase (PAD).

## Abstract 2

Alkene cleaving dioxygenases (ADO), which can oxidatively cleave C=C double bonds to the respective carbonyl compounds, may aid in biocatalytic waste stream utilization strategies by valorizing lignin-derived monomers.

## Graphical abstract

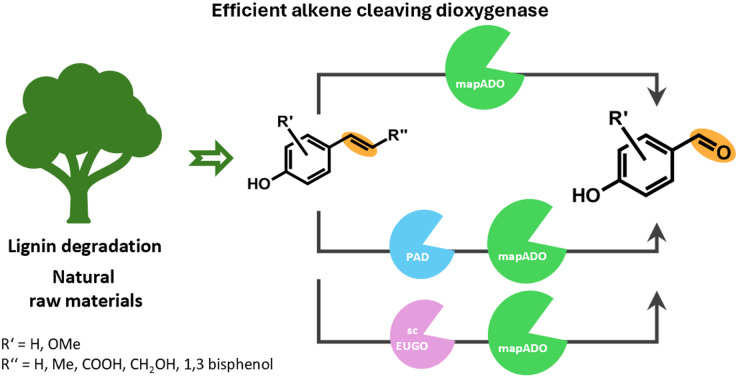

**Green foundation**

1. A newly described enzyme shows unprecedented efficiency in cleaving of alkenes, potentially substituting hazardous processes, and reducing waste.

2. Enzymatic oxidative cleavage of C=C double bonds suffers from low efficiency, while the chemical alternatives require corrosive reagents and/or heavy metal catalysts. The here-described enzyme could be efficient enough to be interesting for industrial applications.

3. Enzyme engineering could further increase the stability and the substrate scope. Alternative carbon sources for the cultivation of the expression host (waste streams or C1-substrates, such as formate or CO_2_) could further reduce the environmental foot-print.

## Introduction

Bio-based substrates and waste streams serve as sustainable sources of raw materials and are gaining increasing importance in the global transition toward a circular economy.^1^ Among these, lignin and cellulose residues—such as those derived from paper pulp—are particularly valuable. These polymers contain diverse aromatic compounds in varying configurations and ratios, which, upon monomerization, can be converted into high-value products for the fragrance, pharmaceutical, and food supplement industries.^2,3^

Especially lignin has gained importance as a potential platform for producing bio-based chemicals and fuels. However, various processing steps are necessary to make lignin available as raw material for valuable compounds. Pretreatment methods, including acidic, organosolv, microbial, and oxidative treatments, are used to separate lignin from lignocellulosic biomass. While the heterogeneity of lignocellulose allows access to many different products, it also results in complex and challenging fractionation processes. The obtained isolated lignin fractions can be further depolymerized by employing, *e.g.*, reducing or oxidizing agents or thermal or acid/base-catalyzed methods. The resulting mixtures of lignin-derived monomers often require additional purification and isolation steps before they can be further converted into high-value products through chemical or biological reactions. Key challenges in this field include high energy consumption, sustainability issues, low yield of desired monomers, reaggregation, and high investment and operating costs.^3–6^ One valorization approach of waste-stream derived compounds is the oxidative cleavage of C=C double bonds to carbonyl compounds.^7^ This can be carried out chemically *via* ozonolysis; however, this method necessitates the handling of highly reactive and potentially hazardous gaseous ozone.^8^ Alternatives include oxidation with hydrogen peroxide or molecular oxygen, which require the presence of catalysts, such as vanadium, but suffer from inefficiency, present challenges in handling, and produce heavy metal pollutants and solvent waste. Enzymatic cleavage of alkenes presents a more environmentally friendly alternative, as it operates under milder conditions with less organic solvent at ambient temperatures and pressures and eliminates the need for heavy metal catalysts. However, enzymatic approaches often suffer from lower conversion rates compared to traditional chemical methods.^9,10^

In this study, we introduce a new alkene cleaving dioxygenase from *Moesziomyces aphidis*, designated as *Map*ADO. Our search for an alternative to the previously studied ‘aromatic dioxygenase’ from *Thermothelomyces thermophilia* (ADO)^9^ which demonstrated suboptimal performance in our laboratory, led us to screen homologous enzymes. Out of 11 promising candidates, *Map*ADO exceeded expectations with remarkable reaction kinetics and conversion efficiency. We characterized the enzyme in detail and identified the primary limiting factor affecting the total yield in the production of vanillin (1c).

**Scheme 1:**
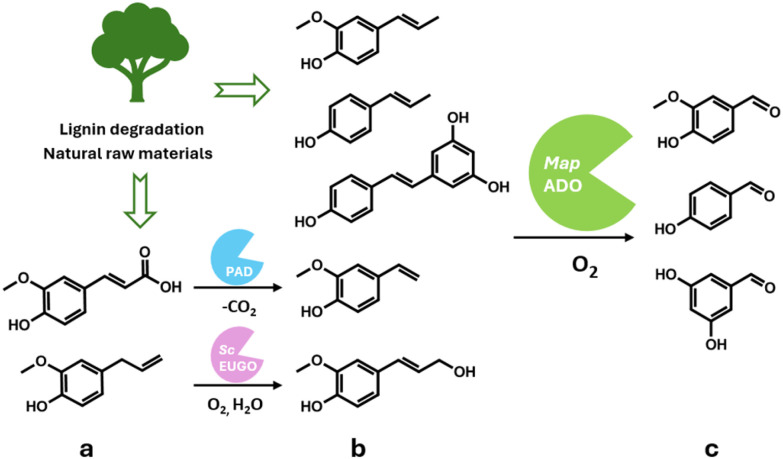
Starting from lignin degradation products or natural raw materials, alkene cleaving dioxygenase *Map*ADO can cleave carbon–carbon double bonds yielding carbonyl compounds without the need for co-enzymes. Upstream reactions with PAD and *Sc*EUGO further enhance the substrate scope.

Further investigation focused on the practical application of *Map*ADO. Isoeugenol (1b) was selected as a substrate for scaling up biocatalysis to the gram scale. Additionally, the substrate scope was expanded by incorporating two upstream enzymatic transformations, targeting key compounds such as ferulic acid (2a)—the major component of lignin waste—and eugenol (3a), commonly found in essential oils. Both compounds were successfully converted into 1c without requiring co-enzymes or co-substrates other than O_2_ (Scheme 1). These findings underscore the potential of *Map*ADO for the valorization of natural raw materials or lignin-derived compounds through alkene cleavage.

## Results and discussion

### Enzyme discovery and substrate scope

**Fig. 1:**
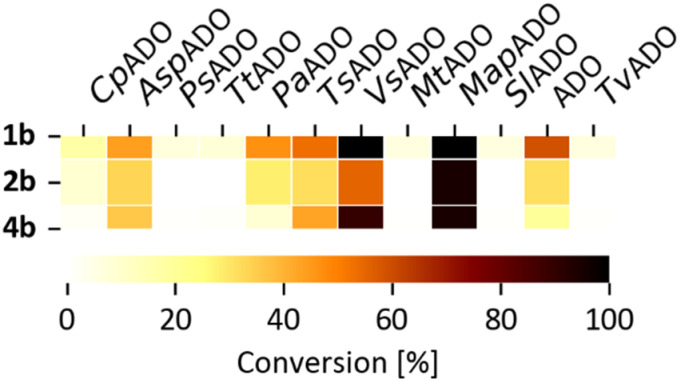
Substrate pre-screening of 11 ADO homologs produced in soluble form in *E. coli* BL21; resting cell biocatalyst; substrate conc: 10 mM. HPLC-MS data of biological triplicates are shown.

Based on similarity search with the ADO sequence as the template,^9^ over 100 hypothetical protein candidates were selected, subjected to phylogenetic analysis (SI Fig. S1) and ranked based on size, essential residues, source of origin and predicted solubility. The most promising sequences (SI Table S3) from fungi, bacteria and plants were chosen based on ranking and position in the phylogenetic tree, to generate utmost diversity. The coding genes were used in a pET-28a vector, expressed in *E. coli* BL21 (DE3) and pre-screened for alkene cleavage activity on 1b, 4-vinyl guaiacol (2b), and hydroxy anethole (4b)(Fig. 1). The best performing enzymes in the initial screening, *Asp*ADO, *Pa*ADO, *Vs*ADO, *Ts*ADO and *Map*ADO were purified and their kinetic parameters with 1b were determined (SI Table S1). Hypothetical protein PaG_05861 from *Moesziomyces aphidis* (*Map*ADO) showed outstanding conversions and showed the highest activity in purified form and was chosen for further investigations. Its substrate scope was further explored with several substituted styrene and stilbene compounds (Table 1). Conversion was observed for 1b, 2b, 3b, 4b and resveratrol (5b), all possessing a hydroxy group in *para*-position to the double bond. The substrate scope indicates that a hydroxy-group in *para*-position is a prerequisite for conversion, which agrees with the proposed mechanism of a protein with highly conserved motifs (NOV1).^11^ This also corresponds with recent investigations on *Map*ADO's crystal structure as well as docking and site-directed mutagenesis studies, which indicate that Y136 and K169 in the active site and the *para*-hydroxy group of the substrate are essential for the positioning of the double bond towards the activated oxygen.^12^ The conversion of isoeugenol acetate (9b) yielded traces of vanillin (1c) instead of vanillin acetate. This suggests a spontaneous hydrolysis of isoeugenol acetate to 1b which is then cleaved to 1c. Conversion of isoeugenol methyl ether (10b) and anethole (7b) were reported for ADO^9^ however, this could not be repeated in our lab, neither with ADO, nor with *Map*ADO or any other homologue that we tested.

**Table 1:** Substrate structures and conversions with *Map*ADO: whole cells (12 g_DCW_ L^−1^) in PBS buffer 10 mM pH 7.4, 10 mM a substrate, 2 v% ethanol, 30 °C, 200 rpm, 24 h, reaction volume: 250 μL, triplicates (SI section 5.3)

	Name	Structure	Conversion [mM] (%)
1b	Isoeugenol	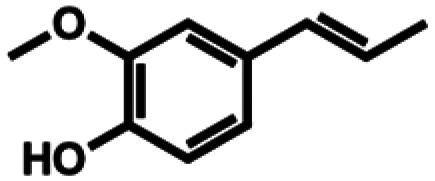	9.55 ± 0.60 (96)
2b	4-Vinylguaiacol	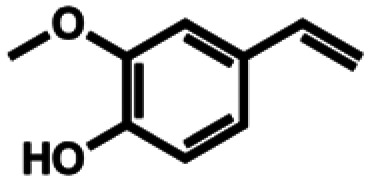	9.52 ± 0.24 (95)
3b	Coniferyl alcohol	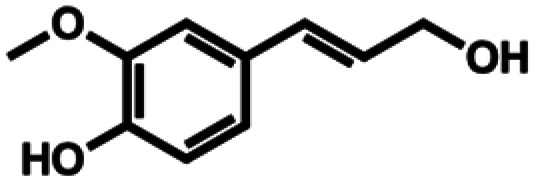	8.92 ± 0.31 (89)
4b	Hydroxy anethole	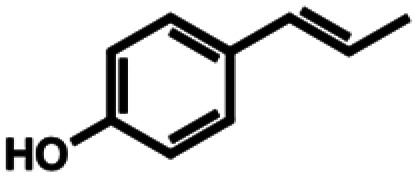	9.52 ± 0.34 (95)
5b	Resveratrol	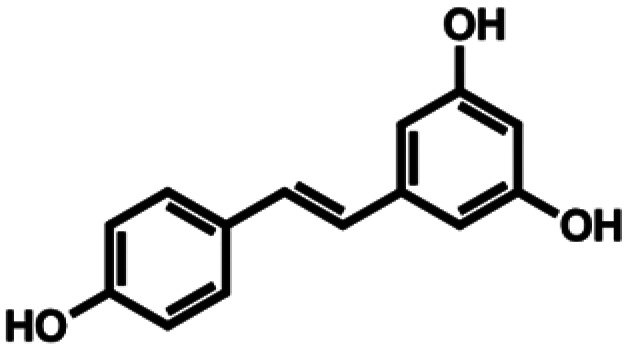	0.56 ± 0.01 (28)
6b	*o*-Isoeugenol	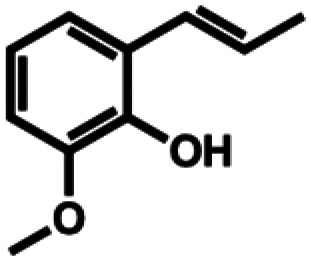	n.c.
2a	Ferulic acid	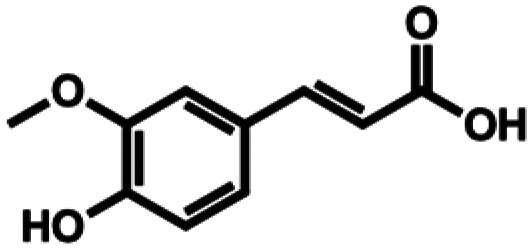	n.c.
3a	Eugenol	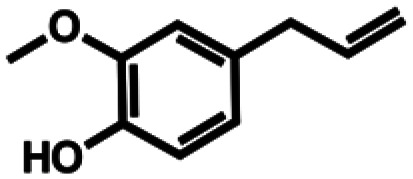	n.c.
7b	Anethole	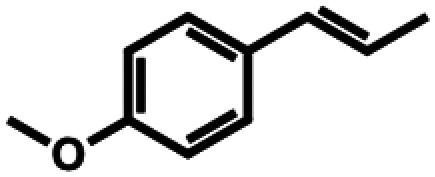	n.c.
8b	3-Vinylanisole	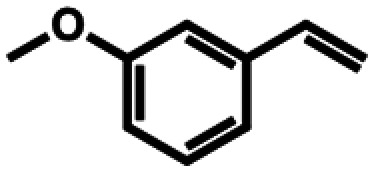	n.c.
9b	Isoeugenol acetate	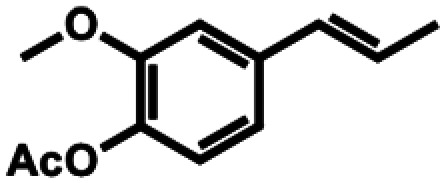	n.c.
10b	Isoeugenol methyl ether	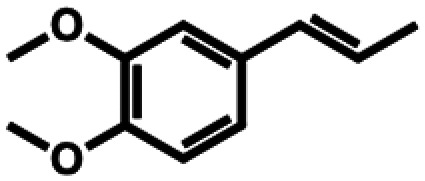	n.c.
11b	Isosafrole	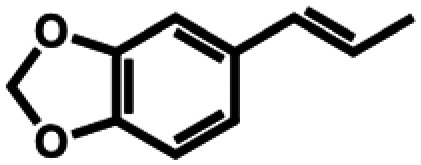	n.c.
12b	Propenyl guaethol	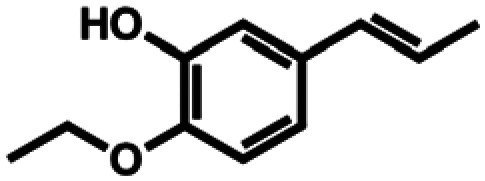	n.c.
13b	β-Methylstyrene	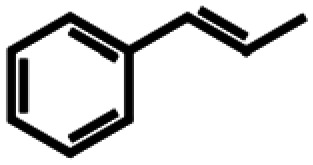	n.c.
14b	3-Methyl-β-methylstyrene	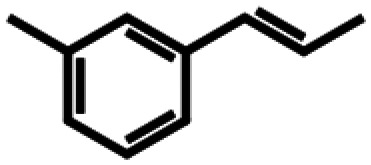	n.c.
15b	4-Methyl-β-methylstyrene	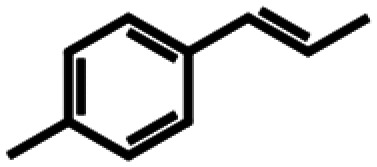	n.c.
16b	α-Methylstyrene	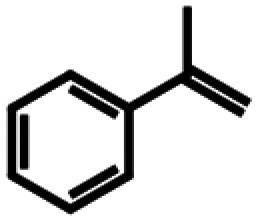	n.c.

a 2 mM 5b were added due to low substrate solubility; n.c. no conversion.

### Enzyme kinetics and biocatalyst characterization

*Map*ADO's outstanding performance is evident from kinetic parameters. Its K_M_ (118 ± 13 μM) and *k*_cat_ (238 ± 2 s^−1^)^12^ were determined through continuous initial rate measurements of vanillin formation based on its absorption. To the extent of our knowledge this is the highest observed activity with 1b among all literature-known enzymes. Similarly, we determined kinetic parameters for 3b. In case of 2b and 4b, spectrophotometric initial rate measurements were hampered due to overlapping absorption of substrate and product. In this case, end point measurements were performed. *Map*ADO exhibited approximately half activity for 2b compared to 1b (Table 2 entry 1) with an outstanding *k*_cat_ of 361 ± 12 s^−1^, that is three orders of magnitude higher than ADO (*k*_cat_ 361 s^−1^*versus* 0.45 s^−1^, respectively)^13^ and other dioxygenases.^14–16^1b is cleaved approximately 3 times faster than 3b, with a *k*_cat_ of 90 ± 8 s^−1^. *Map*ADO shows similar activity for 5b cleavage as NOV1 ^16^ and NOV2.^17^ It is three orders of magnitude faster for 2b.^7^ A direct comparison of kinetic parameters determined by initial rate measurements (Table 2, entry 5) with end-point measurements (Table 2, entry 1) reveals that end-point determination is considerably overestimating both *k*_cat_ and K_M_.

**Table 2:** Comparison of kinetic data of *Map*ADO with selected substrates

Entry	Substrate	K_M_ [μM]	*k*_cat_ [s^−1^]
1	1b a	582 ± 103	654 ± 2
2	2b a	220 ± 46	361 ± 12
3	5b a	49 ± 9	3.2 ± 0.1
4	3b b	445 ± 125	90 ± 8
5	1b b	118 ± 13	238 ± 2

a Endpoint measurement on HPLC.

b Measured on UV-Vis spectrophotometer.

We next explored the operational characteristics of our whole cell biocatalysts. Alkene cleaving activity varied less than 10% in a pH range from pH 6 to 9.0 (SI Fig. S2). Nano differential scanning fluorimetry (NanoDSF) of purified *Map*ADO showed first signs of denaturation at 32.9 °C, with a melting point of 38.2 °C (SI Fig. S3).

This aligns with higher total conversion rates at 20 °C and 30 °C after 24 h as compared to 40 °C. The enzyme was stable for 8 h at 30 °C, but after 24 h of incubation the yield of 1c was reduced by 50% (SI Fig. S4).

While intensifying the reaction for maximal yield of 1c, we observed lower product formation at higher substrate loading. When adding 100 mM 1b with high cell density (12 g_DCW_ L^−1^) of *E. coli* BL21 (DE3) only 30 mM 1c were formed while with 50 mM 1b full conversion was observed. We assumed that substrate or product inhibition might be the reason for the low yield at high substrate concentration.

**Fig. 2:**
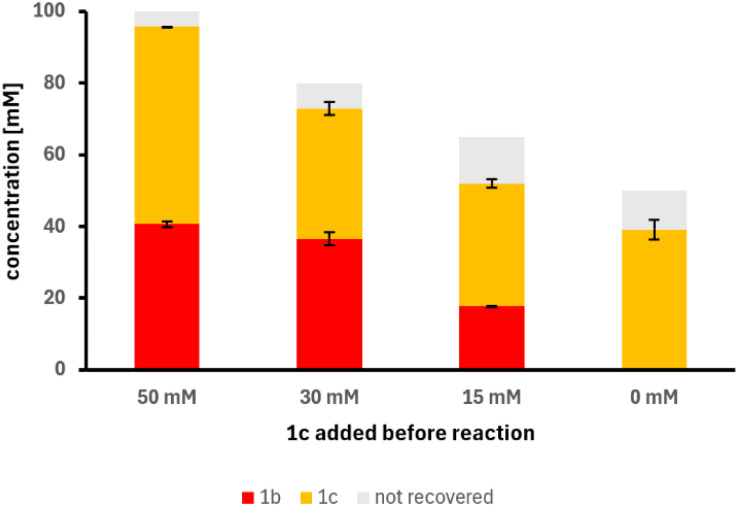
Different amounts of 1c (0, 15, 30, and 50 mM) were added to *Map*ADO cells (12 g_DCW_ L^−1^) with 50 mM 1b. After 24 h at 30 °C, product formation was measured by HPLC. Error bars indicate the standard error of the mean from 2 replicates; grey area shows theoretical yield.

Based on these results we performed experiments with different amounts of 1c spiked to *Map*ADO resting cell biocatalyst, with 50 mM 1b as substrate. When 50 mM of 1c was added together with the substrate, the overall yield was reduced to 10%, with 30 mM of 1c to 13%, with 15 mM of 1c to 39%, while, when no 1c was added beforehand, the same conditions yielded 78% of the desired product 1c (Fig. 2). These results showed product inhibition already at 15 mM of 1c. Notably,^14,18^ vanillyl alcohol or vanillic acid was not observed in any reaction beyond trace amounts. Controls showed that losses in the mass balance were associated to the volatility of isoeugenol. 

### Preparative scale conversion of isoeugenol to vanillin

**Scheme 2:**
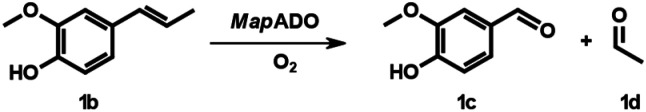
Conversion of isoeugenol to vanillin and acetaldehyde.

**Fig. 3:**
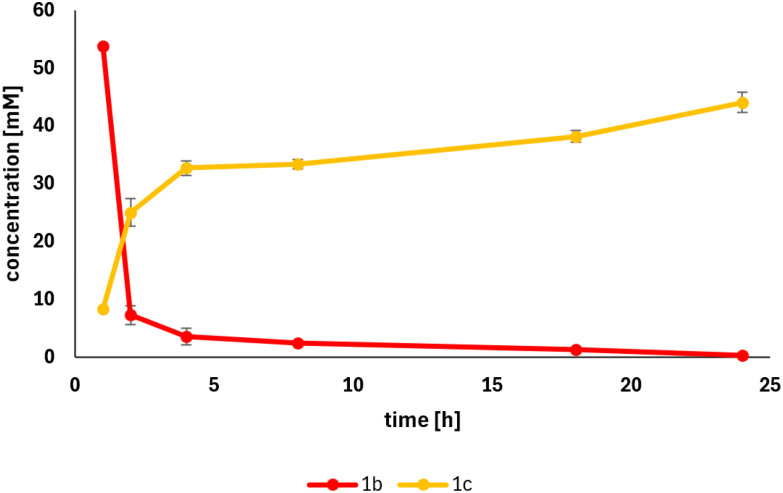
Preparative scale conversion of isoeugenol to vanillin; error bars were calculated based on triplicate samples taken from the reaction mixture. Initial substrate concentration: 50 mM; biocatalyst loading: 4 g_DCW_ L^−1^.

With this inhibition limitation in mind, we applied *Map*ADO for bio-vanillin production at a preparative scale using 50 mM 1b (Scheme 2) and achieved over 99% conversion, a final concentration of 6.69 g L^−1^ after 24 h (Fig. 3) and 1.29 g of 1c were isolated by extraction with ethyl acetate. The product required no further purification *e.g.* by chromatography, and its identity and purity were confirmed by NMR analysis.

In comparison, preparative scale reactions of 1b to 1c, using isoeugenol monooxygenase (IEM) from *Pseudomonas nitroreducens* in combination with sol–gel chitosan membranes for product removal reported 29.6 mM or 4.5 g L^−1^1c with a conversion rate of 75% and a space–time yield of 3.6 g L^−1^ d^−1^, respectively.^19^ Other sources used a similar system to increase the conversion to 84% and isolated 1.127 g 1c, giving an isolated yield of 82.3%.^20^ The simple scale-up presented in this work achieved an isolated yield of 85% corresponding to a space–time yield of 6.45 g L^−1^ d^−1^ without employing any feeding or product removal strategies, surpassing previous reports.

### Cascade: eugenol to vanillin

**Scheme 3:**
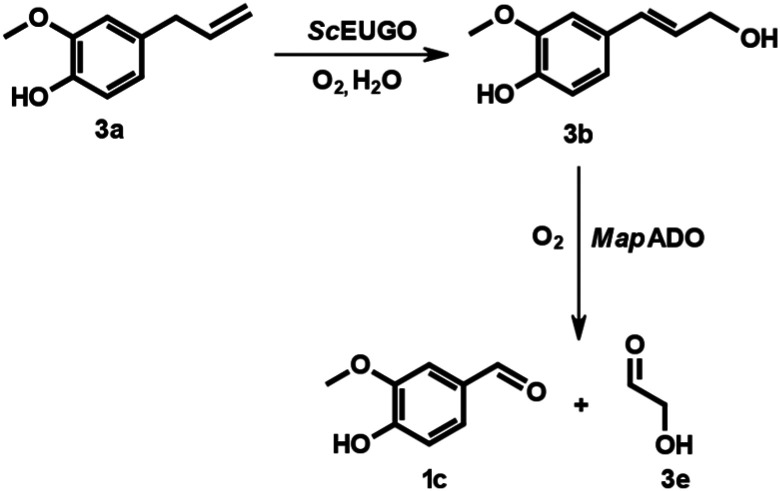
Conversion of eugenol to vanillin and glycolaldehyde with coniferyl alcohol as intermediate.

The isomer of 1b – eugenol (3a) – is a renewable phenylpropene that can be extracted from clove, cinnamon or nutmeg.^21^ We explored an enzymatic cascade, with eugenol oxidase *Sc*EUGO^22^ for isomerization of the double bond to give 3b followed by *Map*ADO mediated C–C double bond cleavage^13^ (Scheme 3). This two-step reaction allowed using 3a directly as a substrate without the need for a preceding isomerisation into 1b, which is usually done *via* metal catalysis.^23^ In the course of our study, the same concept was explored by Kroutil *et al.* using *Rj*EUGO and a mutant dioxygenase denoted ACO-03 C26N.^18^ For our cascade experiment, whole cells with *Map*ADO and *Sc*EUGO were mixed in a vial and shaken at 30 °C, at 200 rpm for 24 h.

**Fig. 4:**
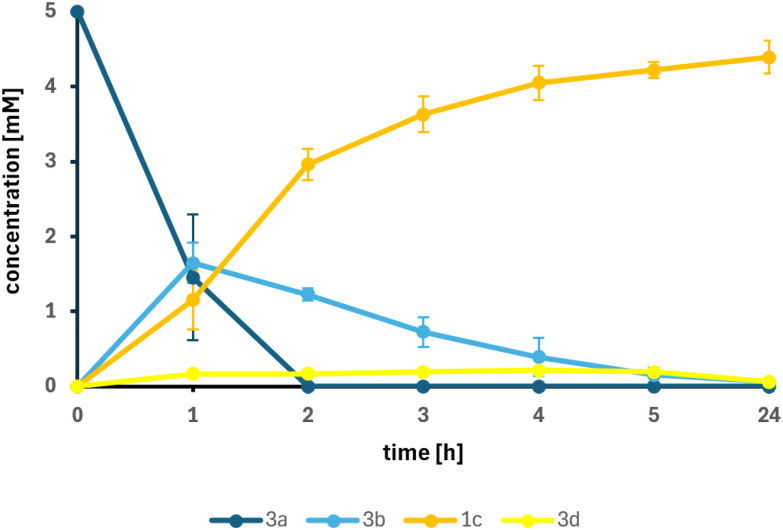
Time-resolved cascade reaction using 5 mM 3a with *Sc*EUGO whole cells (1 g_DCW_ L^−1^) and *Map*ADO whole cells (12 g_DCW_ L^−1^) in PBS buffer 10 mM pH 7.4, 2 v% ethanol, at 30 °C and 200 rpm, reaction volume: 250 μL. Error bars indicate the standard error of the mean from 3 replicates.

Several experiments with varying substrate concentrations and cell densities were tested. The data showed that 5 mM 3a was completely converted to 1c using *Sc*EUGO with 1 g_DCW_ L^−1^ and *Map*ADO with 4 g_DCW_ L^−1^. When the biomass concentration of *Map*ADO expressing cells was increased to 12 g_DCW_ L^−1^, even 10 mM 3a were converted completely. Using 15 mM 3a resulted in 8.86 mM 1c and some traces of unconverted 3b (Table 3). The addition of catalase did not increase overall conversion, eliminating released H_2_O_2_ as limiting factor (SI Fig. S5). Time-resolved experiments were conducted to determine when 3a and 3b were completely consumed (Fig. 4). Traces of coniferyl aldehyde (3d) were detected as a side-product, likely caused by endogenous genes from the *E. coli* strain used.^18,24^ Attempts to increase the 3a conversion by a fed-batch approach where 3a was added every 2 h or 5 h did not increase the yield. Inhibition by glycolaldehyde (3e) or oxygen limitation were excluded experimentally (see SI Fig. S6 and S7).

**Table 3:** Results of the cascade reaction using varying substrate concentrations and different amounts of *Map*ADO catalyst

Start conc. 3a [mM]	*Map*ADO g_DCW_ L^−1^	3a [mM]	3b [mM]	1c [mM]
5	12	0	0	4.25 ± 0.05
5	8	0	0	3.48 ± 0.49
5	4	0	0	4.05 ± 0.41
5	1	0	0.71 ± 0.08	3.33 ± 0.31
10	12	0	0	7.62 ± 0.37
15	12	0	1.90 ± 0.14	8.86 ± 0.41

Similar enzymatic reactions have been described before and identified oxygen supply as a limiting factor, for example, in the *Rj*EUGO – ACO-03 C26N cascade, which was conducted as a sequential reaction sequence. The alkene cleavage step exhibited optimal efficiency under conditions of O_2_ bubbling and stirring, necessitating the presence of catalase, ascorbate, and dithiothreitol (DTT), along with regular supplementation of FeSO_4_.^18^

A comparison of the green metrics of this biotransformation and the cascade published by Lanfranchi *et al.* can be found in the SI (chapter 6). Other reported strategies for converting 3a to 1c include biotransformations employing bacterial strains^25,26^ or fungal species such as *Daldinia* sp. IIIMF4010.^27^ In the latter case, starting from 50 mM 3a, the reaction yielded 3.1 mM (6.2%) eugenol-β-d-glucopyranoside and 10.9 mM (21.8%) 1c. The reaction pathway presented in this study offers the advantage of achieving complete conversion to 1c at low eugenol concentrations, thereby eliminating the need for side-product separation. Recently, Zhu *et al.* developed an artificial biosynthetic pathway incorporating three enzymes to convert 3a to feruloyl-CoA, which can subsequently be transformed into 1c.^28^ After optimization, this approach yielded high 1c production, reaching 4.83 ± 0.09 g L^−1^ (88.2% yield). Key enhancements included *in vivo* directed evolution of the rate-limiting enzyme, a cofactor switch from NADP^+^ to NAD^+^, inactivation of endogenous aldehyde reductases, and sequential eugenol supplementation.^28^ While the two-step cascade described herein achieved comparatively lower yields, further optimization of the process and enzyme engineering could potentially lead to higher product concentrations, especially as the cascade proposed in the work does not require NAD(P)H or acetyl-CoA.

### Cascade: ferulic acid to vanillin

**Scheme 4:**
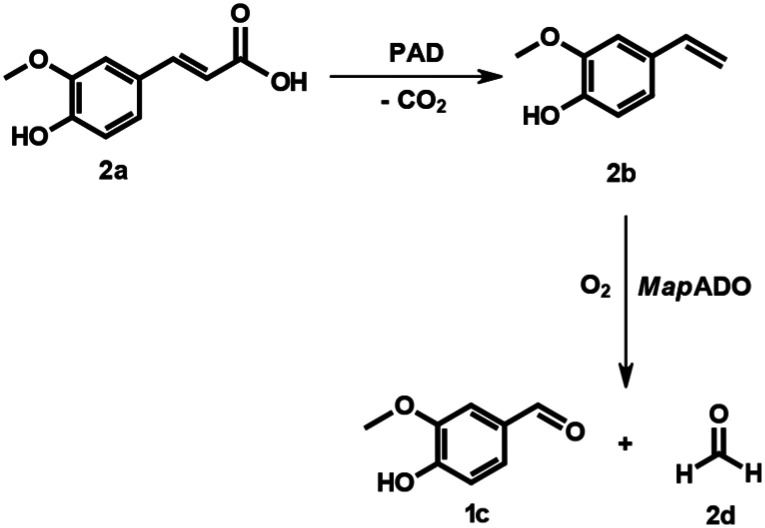
Conversion of ferulic acid to vanillin and formaldehyde with 4-vinyl guaiacol as intermediate.

**Fig. 5:**
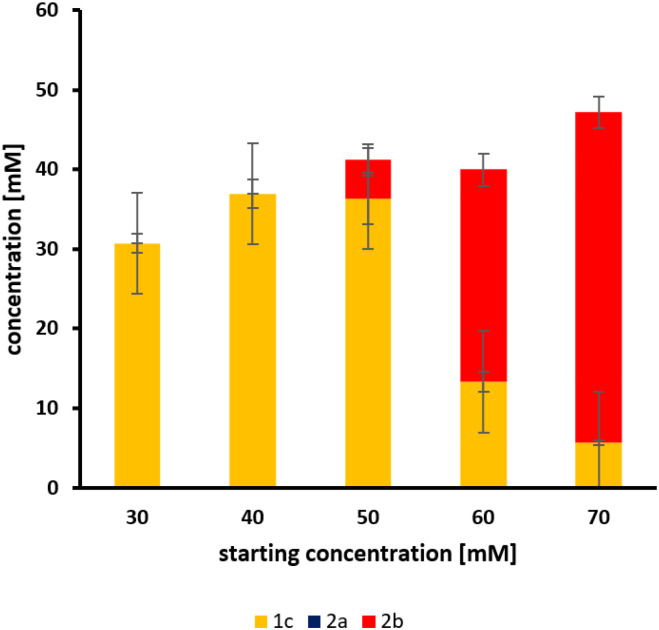
Whole-cell biotransformations of 2a to 1c with PAD and *Map*ADO. Both enzymes were simultaneously expressed in *E. coli* BL21 (DE3) and suspended to a cell density of 12 g_DCW_ L^−1^ in 200 mM phosphate buffer. 2a was added from 2 M stock solution in DMSO. Samples were taken after 24 h and error bars indicate the standard deviation of the mean from 3 replicates.

In the second cascade reaction, ferulic acid, a major monomer in lignin, was used as a substrate, following the seminal concept of Kino *et al.*^29,30^ This required the decarboxylation of the substrate, which was done by a phenolic acid decarboxylase (PAD) from *Bacillus coagulans* DSM11 (Scheme 4). Both enzymes were expressed in *E. coli* BL21 (DE3) on a single plasmid. The decarboxylation was effective and resulted in the rapid accumulation of 2b, which was subsequently converted into 1c. The full conversion of up to 40 mM 2a to 1c was successful, while at higher concentrations, 2b accumulated (Fig. 5). It should also be mentioned that this reaction was pH-sensitive and required a higher buffer concentration (200 mM phosphate buffer, pH 7.4).

Previous studies have reported final product concentrations of up to 8.0 mM for a one-pot reaction with a cofactor-free enzymatic cascade.^29^ In contrast, the two-enzyme cascade presented in this work achieved product concentrations of up to 36.9 mM. Subsequent optimization increased product yield to 52 mM 1c; however, this approach required two separate reactions conducted at high pH (9.0 and 10.5).^28^ In comparison, the cascade described with *Map*ADO is highly active at a pH of 7.4. *Map*ADO could potentially be tailored towards one-step production of vanillin as shown recently for ADO.^24^

## Conclusions

This study successfully identified several new alkene cleaving dioxygenases, and characterized the most promising candidate, *Map*ADO from *Moesziomyces aphidis*, demonstrating its efficiency in alkene cleaving of lignin-derived substrates such as 1b and 2b. The enzyme exhibits superior kinetic properties not only for isoeugenol but also for 4-vinylguaiacol, resulting in a hundred-fold higher specific activity *k*_cat_ of 361 *versus* 4.4 s^−1^.^14^*Map*ADO's activity and stability render it a promising candidate for the sustainable synthesis of bio-vanillin. Here we showed convergent examples by converting three different precursors to vanillin. Components of lignin waste streams (2a) or bio-available compounds (3a) are substrates for *Map*ADO with simple reactions that basically only require pH control and O_2_. Product inhibition has been identified as the main limiting factor, which in future studies should be tackled by process engineering measures such as *in situ* product removal. These findings contribute to the expanding enzymological toolbox for valorising waste streams.

## Experimental

### General

Standard reagents were obtained from VWR, Sigma-Aldrich (Vienna, Austria) or Roth GmbH & Co. KG (Karlsruhe, Germany). Restriction enzymes were obtained from Thermo Scientific (St Leon Rot, Germany).

### Strains and plasmids

*Escherichia coli* Top 10F′ or *E. coli* Top 10 was used for molecular cloning and plasmid propagation. *E. coli* BL21 (DE3) was used for the expression of recombinant proteins. Codon-optimized sequences of 11 hypothetical proteins (SI Table S3) were cloned into the vector pET-28a and expressed in *E. coli*. ADOs were equipped with His ×6 affinity tags for purification. ADOs were expressed in *E. coli* BL21 (DE3) using LB medium, with gene expression induced at 20 °C by addition of 1 mM of isopropyl β-d-1-thiogalactopyranoside (IPTG) and supplementation with 1 mM of FeCl_2_. Detailed methods for cloning, expression, and purification are provided in the SI.

### Biotransformation reactions with *Map*ADO

Biotransformation reactions were performed in potassium phosphate buffer 10 mM pH 7.4 with resting cells at varying dry cell weights. The substrates were added as stock solutions in DMSO or ethanol. The final amount of co-solvent in the reaction was 2 v% ethanol or DMSO, respectively. The reactions were done in 4 mL brown-glass vials with screw-caps or 12 mL Pyrex glass tubes and were incubated at 30 °C or 40 °C on a tissue cultivation rotor, a thermoshaker or incubator shaker. For analytical measurements, samples were drawn from the reaction mixture or the entire reaction mixture was utilized. Samples were either extracted twice with an equal volume of ethyl acetate containing acetophenone (10 mM) as internal standard and were measured on HPLC. Alternatively, the reaction mixture was diluted with acetonitrile (depending on the concentration 1 : 2, 1 : 7 or 1 : 15) and centrifuged (16 162*g*, rt, 2 min, SIGMA 1–14 Microfuge). The supernatant was then filtered into an HPLC vial *via* syringe filter or transferred to Syringeless Filter Vials (SEPARA®).

### HPLC-UV analysis

For HPLC-UV measurements two devices were used. Kinetic data, measurements for the preparative scale and data for Fig. 3 and SI Fig. S2 were analyzed as follows: Samples were analysed using an HPLC-UV on an LC-MS 2020 system equipped with a UV detector (Shimadzu). The utilized column was an EC 150/3 Nucleodur C18 Gravity (3 μm, Macherey-Nagel, REF 760083.30). The mobile phases were 0.1% formic acid in double-distilled water and acetonitrile. Detection was performed at 280 nm with the temperature maintained at 40 °C and a flow rate of 1 mL min^−1^. The solvent gradient was: 10% ACN from 0–0.7 min, 10–90% ACN from 0.7–3.1 min, 90% ACN from 3.1–3.8 min, 10% ACN from 3.8–5.0 min. Data were evaluated using LabSolution software (Shimadzu).

All other samples were measured on a Shimadzu Nexera HPLC device with LC-40D XR pumps, CTO-40C column oven, SIL-40C XR autosampler, CBM-40 system controller, DGU-405 degasser and SPD-M40 photodiode array detector. The measurements were conducted on a C18 column (Waters XSelect CSH C18 XP column, 130 Å, 3.5 μm, 3.0 × 50 mm) at an oven temperature of 40 °C, with a flow rate of 1.3 mL min^−1^. A gradient of HPLC-grade acetonitrile and HPLC-grade water was used for the elution. For the acidic method, 0.1 vol% formic acid was added to the eluent, for the basic method, an ammonium acetate solution (2.5 mM, pH = 8.5) was used. The solvent gradient for the acidic method was: 5% ACN until 0.15 min, 5–95% ACN from 0.15–2.5 min, 95% ACN from 2.5–2.8 min, 95%–5% ACN from 2.8–3.0 min, 5% ACN from 3.0–3.3 min. The solvent gradient for the basic method was: 5% ACN until 0.15 min, 5–60% ACN from 0.15–2.5 min, 60% ACN from 2.5–2.8 min, 60%–5% ACN from 2.8–3.0 min, 5% ACN from 3.0–3.3 min. For detection, the PDA peaks at 260 nm, 270 nm or 254 nm were analyzed. The quantification of the compounds was done using calibration curves with authentic standards.

### Inhibition experiments

For testing 1c inhibition, 4 mL brown-glass vials with screw caps were charged with 250 μL *Map*ADO whole cells (12 g_DCW_ L^−1^) in PBS buffer 10 mM pH 7.4, ethanol (2% v/v), and 50 mM 1b. Furthermore, various amounts of 1c (0 mM, 15 mM, 30 mM, and 50 mM) were added to the vials. The vials were shaken at 30 °C at 200 rpm. After 24 h, the reaction mixtures were diluted with HPLC-grade acetonitrile, centrifuged, filtered, and measured on HPLC.

For 3e inhibition experiments, 4 mL brown-glass vials with screw caps were charged with *Map*ADO whole cells (12 g_DCW_ L^−1^) and *Sc*EUGO whole cells (1 g_DCW_ L^−1^) suspended in PBS buffer 10 mM pH 7.4. 10 mM 3a (1 M stock solution in ethanol), ethanol (2% v/v), and various amounts of glycolaldehyde (as dimer; 0 mM, 2.5 mM, 5 mM, and 10 mM) were added (SI Fig. S6). The total reaction volume was 250 μL. The closed vial was shaken at 30 °C at 200 rpm for 24 h. Workup was done as described above.

### Spectrophotometric assay for determination of kinetic parameters

Kinetic curves with 1b, 2b or 5b were determined in reactions containing potassium phosphate buffer (10 mM, pH 7.4), IMAC purified enzyme (0.4 mg mL^−1^), substrate (0.05 mM to 2 mM), and EtOH (2% v/v) in a total volume of 200 μL. Reactions were carried out in triplicates of biological triplicates. The increase in absorbance, corresponding to vanillin formation, was monitored at 350 nm for 1 min at 30 °C using a Synergy Mx Platereader (Biotek).

### Determination of kinetic parameters using endpoint measurements with HPLC-UV

Kinetic curves with 1b or 3b, were determined as endpoint measurements in reactions containing potassium phosphate buffer (10 mM, pH 7.4), IMAC purified enzyme (0.2 to 1.0 mg mL^−1^), substrate (0.02 mM to 6 mM), and EtOH (2% v/v) in a total volume of 200 μL. Reactions were carried out in technical triplicates at 30 °C. Reactions were stopped and extracted twice with an equal volume of EtOAc containing acetophenone (10 mM) as an internal standard after 20 min and measured on LC-MS 2020 system (Shimadzu).

### Preparative scale conversion of isoeugenol to vanillin

Preparative scale reactions (200 mL total volume) contained 50 mM 1b, 2 vol% EtOH, and resting cell suspensions (4 g_DCW_ L^−1^) in 10 mM potassium phosphate buffer (pH 7.4). Reactions were incubated at 30 °C and 130 rpm for 24 h. Following incubation, cells were separated by centrifugation (15 min, 4 °C, 14 000*g*). The buffer phase was acidified with concentrated HCl and extracted with ethyl acetate until TLC (4 : 1 light petroleum : ethyl acetate) confirmed complete extraction. The organic layers were combined, washed with brine, dried over Na_2_SO_4_, and concentrated by rotary evaporation. 1.29 g (85%) of vanillin as slightly beige solid was obtained. An NMR spectrum of the product was measured (see SI). The spectrum is in accordance with literature.^31^

### Enzyme cascade of eugenol to vanillin

*Sc*EUGO was produced as described,^22^ with modifications outlined in the SI. The cascade reactions were performed in 4 mL brown-glass vials with screw caps. The vial was charged with *Map*ADO whole cells (12 g_DCW_ L^−1^) and *Sc*EUGO whole cells (1 g_DCW_ L^−1^) suspended in PBS buffer 10 mM pH 7.4. Then, the respective amount of 3a (1 M stock solution in ethanol) and 2% v/v of ethanol as co-solvent were added. The total reaction volume was 250 μL. The closed vial was shaken at 30 °C at 200 rpm for 24 h. Afterwards, the reaction mixture was diluted with HPLC-grade acetonitrile (1 : 4), centrifuged, filtered, and measured on HPLC.

### Enzyme cascade of ferulic acid to vanillin

The conversion of 2a to 1c required the additional expression of a phenolic acid decarboxylase (PAD). The PAD from *Bacillus coagulans* DSM11,^9^ was chosen. PAD and *Map*ADO were cloned into one pET28-vector and enzyme expression was done as described above. The cells were harvested by centrifugation and resuspended in a 200 mM phosphate buffer (pH 7.4) to an OD_590_ ∼ 60. The reaction was done by using 1 mL cell suspension in an 8 mL glass vial at 30 °C and 200 rpm. Samples were diluted 1 : 15 or 1 : 40 in acetonitrile and analyzed *via* UHPLC. It should be mentioned, that the reaction was highly pH sensitive and lower buffer concentrations strongly reduced the yield.

## Author contributions

F. R. and M. W.: conceptualization, funding acquisition, project administration, supervision, writing-original draft, writing-review & editing; A. S.: investigation, visualization, writing-original draft, writing-review & editing; L. S.: investigation, writing-original draft, writing-review & editing; T. R.: investigation, visualization, writing-original draft.

## Conflicts of interest

There are no conflicts to declare.

Data availability

Supplementary information is available. Additional experimental data, NMR spectra and Green Metrics can be found in the SI. See DOI: https://doi.org/10.1039/d5gc01848j.

## Supplementary Material

GC-027-D5GC01848J-s001
