# Supplementary material for: Co-enzyme-free, enzymatic synthesis of aldehydes from renewable resources with a new and highly efficient alkene cleaving dioxygenase
Source: Green Chem. 2025 Aug 4;27(34):10234–41. doi: 10.1039/d5gc01848j (PMC12333492; doi:10.1039/d5gc01848j)
Supplement: GC-027-D5GC01848J-s001 [file GC-027-D5GC01848J-s001.pdf]

Supplementary Information (SI) for Green Chemistry.
This journal is © The Royal Society of Chemistry 2025

Supplementary Information

Co-enzyme-free, enzymatic synthesis of aldehydes from renewable resources with a new, highly efficient alkene cleaving dioxygenase

Astrid Schiefer[1] Lukas Schober[2], Thomas Rohr[1], Margit Winkler[2,3,*], Florian Rudroff[1,3,*]

[1]Institute of Applied Synthetic Chemistry, TU Wien, Getreidemarkt 9, 1060 Vienna, Austria
[2]Institute of Molecular Biotechnology, Graz University of Technology, NAWI Graz, Petersgasse 14, Graz, Austria.
[3]Austrian Center of Industrial Biotechnology, Krenngasse 37, Graz, Austria

Table of Contents

1. Supplementary figures and tables 3

1.1 Phylogenetic tree 3

1.2 pH profile of *Map*ADO whole cells biocatalyst based on conversion of **1b** 4

1.3 T_m determination of *Map*ADO by nanoDSF 4

1.4 Stability experiments of *Map*ADO at 30 °C 5

1.5 Cascade reaction with *Sc*EUGO and *Map*ADO: addition of catalase 5

1.6 Glycolaldehyde inhibition test of cascade reaction with *Sc*EUGO and *Map*ADO 6

1.7 Oxygen measurements during the cascade reaction with *Sc*EUGO and *Map*ADO 6

1.8 Kinetic data of selected alkene cleaving dioxygenases with **1b** 7

2. Strains & Plasmids 7

3. Gene Sequences 9

3.1 Sequence of *Ps*ADO 9

3.2 Sequence of *Tv*ADO 10

3.3 Sequence of *Ts*ADO 10

3.4 Sequence of *Map*ADO 11

3.5 Sequence of *Asp*ADO 11

3.6 Sequence of *Pa*ADO 12

3.7 Sequence of *Mt*ADO 12

3.8 Sequence of *Tt*ADO 13

3.9 Sequence of *Sl*ADO 13

3.10 Sequence of *Vs*ADO 14

3.11 Sequence of *Cp*ADO 14

3.12 Sequence of eugenol oxidase (*Sc*EUGO) from *Streptomyces cavernae* 15

4. Methods 16

4.1 Preparation of ADO plasmids 16

4.2 Enzyme expression 16

4.3 Enzyme purification 17

4.4 Kinetics 17

4.5 Buffer and media 18

5. Analytics 19

5.1 SDS-PAGE 19

5.2 HPLC 20

5.2 NMR 25

6. Green Metrics 32

References 33

1. Supplementary figures and tables

1.1 Phylogenetic tree

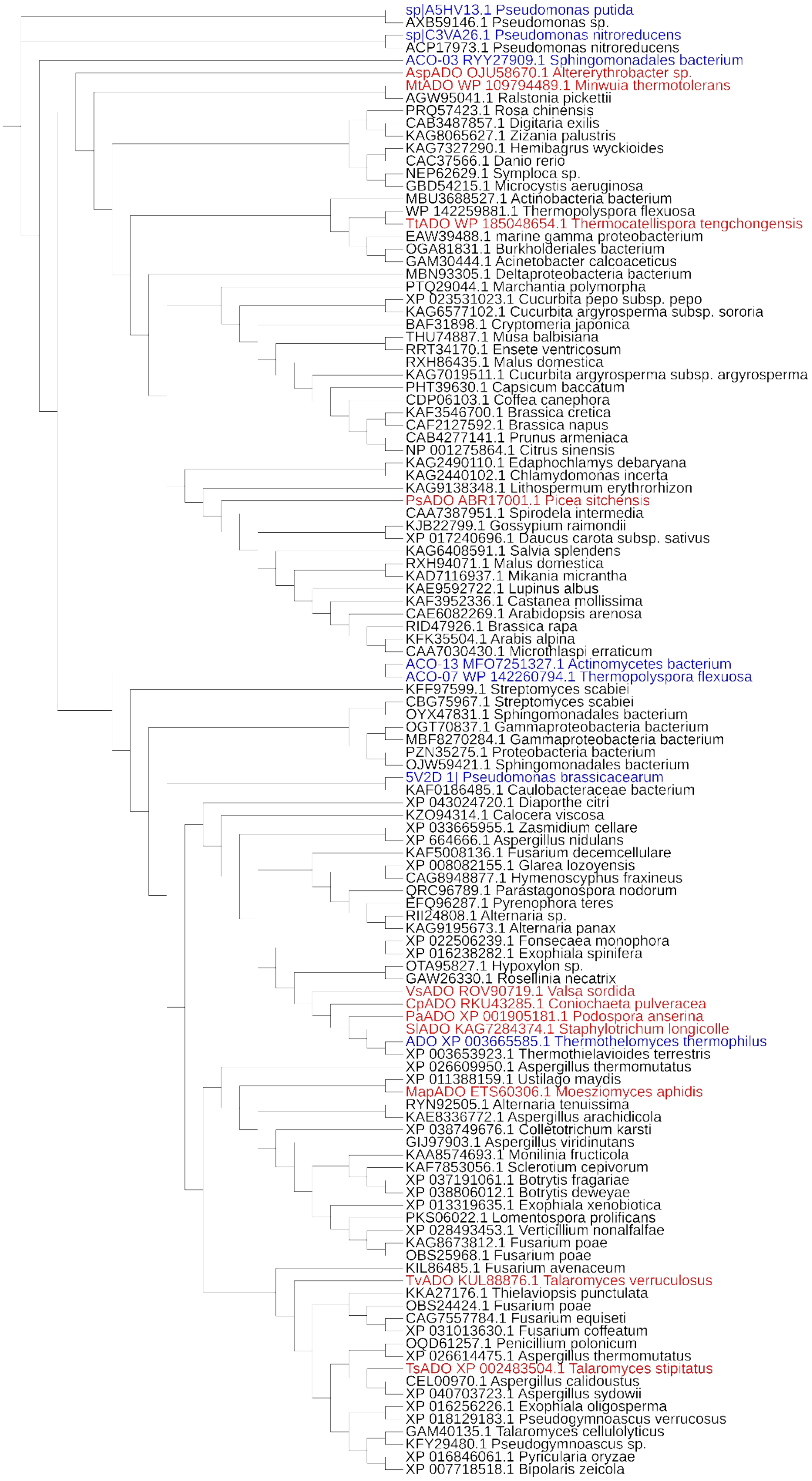

Figure S1: Phylogenetic tree of several CCOs (Black: putative CCOs; blue: literature known CCOs; red: CCOs explored in this work)

1.2 pH profile of *Map*ADO whole cells biocatalyst based on conversion of **1b**

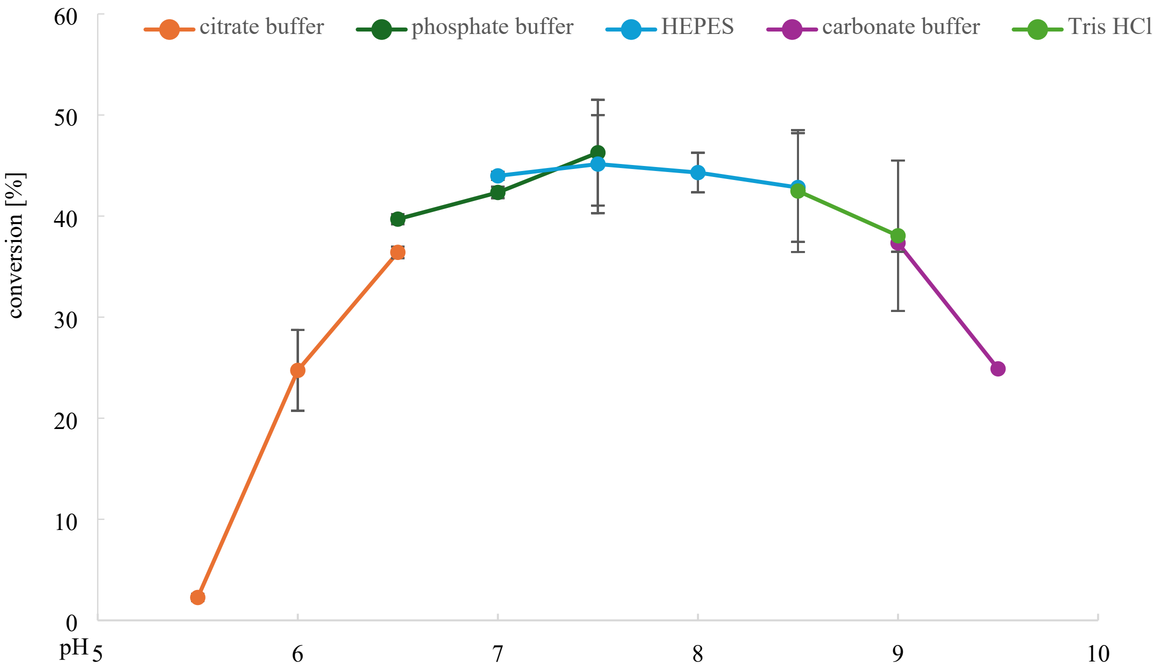

*Figure S2: Conversion of **1b** to **1c** with MapADO in whole cells (2 g_{DCW} L^{-1}), 20 mM substrate; reaction time 30 min; reaction temperature: 40 °C; all reactions were prepared in triplicates.*

1.3 T_m determination of *Map*ADO by nanoDSF

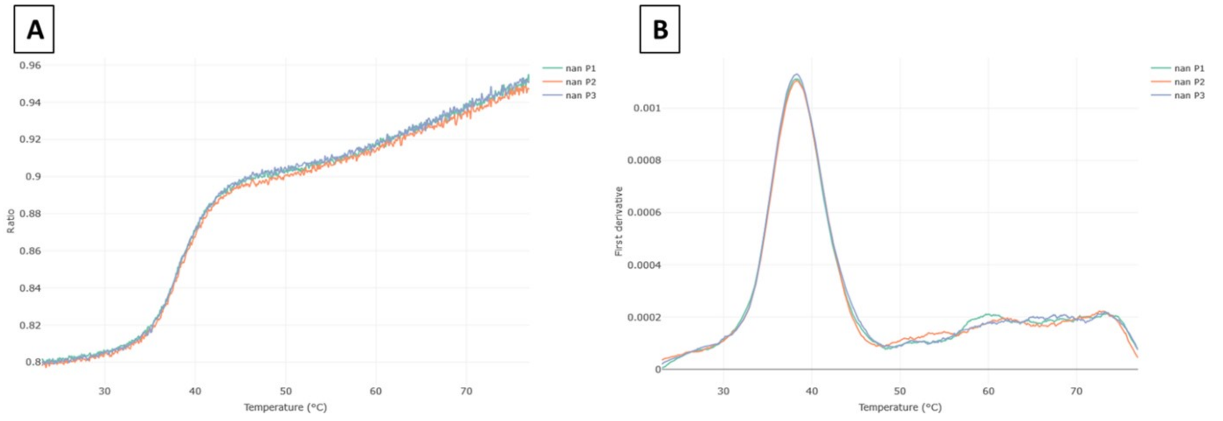

*Figure S3: Temperature-dependent denaturing curve of MapADO recorded with Nano Differential Scanning Fluorimetry. MapADO was expressed in E. coli BL21 and isolated via immobilized metal affinity chromatography. 1 mg mL^{-1} protein was dissolved in PBS buffer (pH 7.4). Temperature increased 0.1 °C min^{-1} from 20 to 80 °C. **A:** y-axis shows the ratio of fluorescence at 330 nm and 350 nm. **B:** shows the derivative of graph A. The experiment was done in triplicates, each color representing one technical replicate.*

1.4 Stability experiments of *Map*ADO at 30 °C

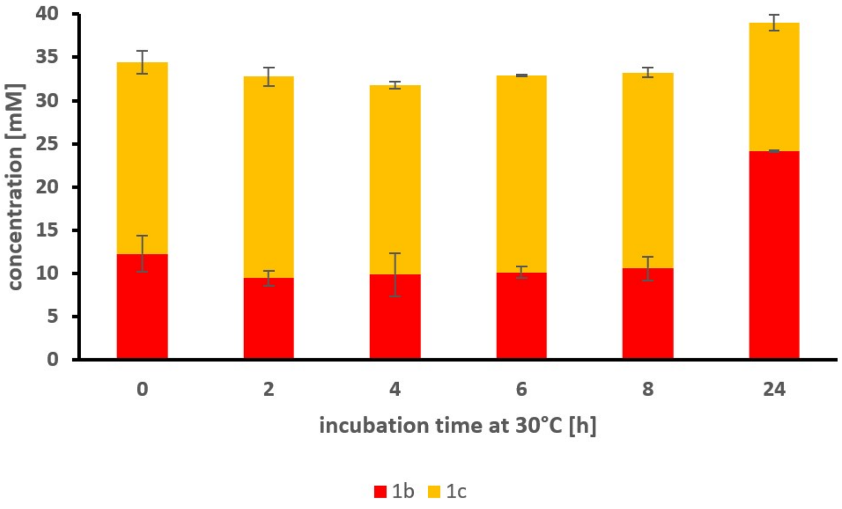

*Figure S4: Stability of MapADO at 30° C. E. coli BL21 cells, expressing MapADO, were incubated at 30 °C with a cell density of 6 g_{DCW} L^{-1}. After 0, 2, 4, 6, 8, and 24 h, 40 mM **1b** was added and 60 min later, the concentration of **1b** and **1c** was measured on HPLC. Error bars indicate the standard error of the mean from 2 biological replicates.*

1.5 Cascade reaction with *Sc*EUGO and *Map*ADO: addition of catalase

H_2O_2 is a by-product of *Sc*EUGO oxidation and may lead to catalyst inactivation. *E. coli* whole cell catalysts do harbor catalase. Nevertheless, we examined catalase (15000 U/mL) addition[1] to the cascade reaction. Bovine serum albumin (bsa) was added to the respective control reactions instead of catalase. The reactions with additional catalase and bovine serum albumin showed no significant difference in the outcome so it can be concluded that sufficient catalase from *E. coli* is in the reaction mixture.

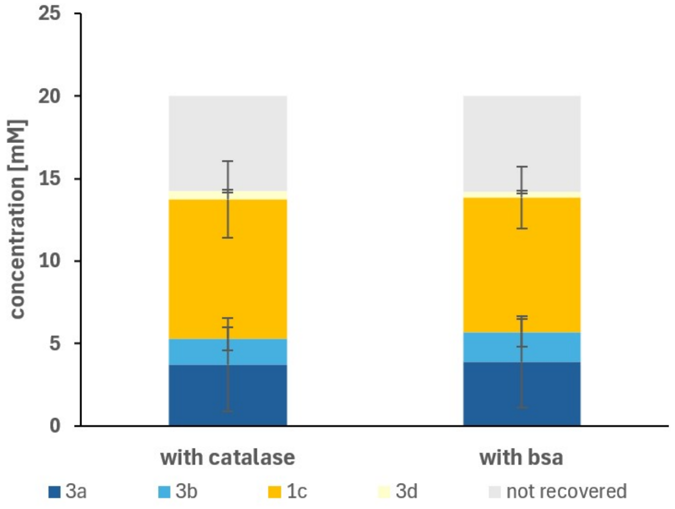

*Figure S5: Effect of catalase addition. 20 mM **3a**, ScEUGO whole cells (1 g_{DCW} L^{-1}), MapADO whole cells (12 g_{DCW} L^{-1}), catalase 15000 U/mL or bovine serum albumin, in PBS buffer 10 mM pH 7.4, at 30 °C and 200 rpm for 24 h 2 % v/v ethanol, reaction volume: 250 µL, biological triplicates, HPLC data.*

1.6 Glycolaldehyde inhibition test of cascade reaction with *Sc*EUGO and *Map*ADO

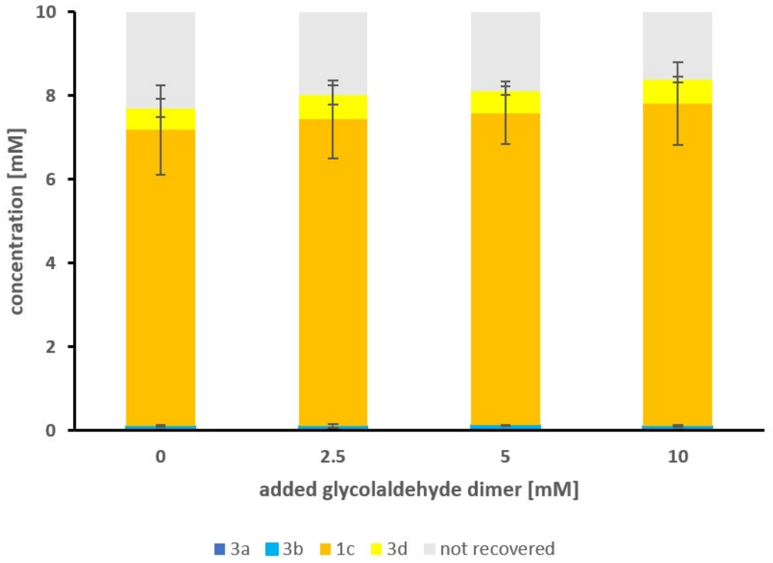

Figure S6: Exploration of potential inhibition by side product of alkene cleavage. 10 mM ***3a****, ScEUGO whole cells (1* g_{DCW} L^{-1}*), MapADO whole cells (12* g_{DCW} L^{-1}*), in PBS buffer 10 mM pH 7.4, at 30 °C and 200 rpm for 24 h, 2 % v/v ethanol, reaction volume: 250 µL, biological triplicates, HPLC data.*

1.7 Oxygen measurements during the cascade reaction with *Sc*EUGO and *Map*ADO

Oxygen measurements during the cascade reaction with *Map*ADO and *Sc*EUGO were performed to test if the reaction is limited by the lack of oxygen. For this experiment, three oxygen sensors (PyroScience GmbH, OXROB10) were used. 8 mL glass vials were charged with 1 mL of *Map*ADO whole cells (12 g_{DCW} L^{-1}) and *Sc*EUGO whole cells (1 g_{DCW} L^{-1}) suspended in PBS buffer 10 mM pH 7.4. The oxygen sensors were dipped into the suspension. 15 mM of **3a** were added via a syringe. The vials were shaken at 30 °C at 200 rpm for 24 h. 8 mL vials were used as reaction vessels because the sensors could be mounted nicely. 1 mL reaction suspension was used for this test because the sensors need a certain volume of liquid to always be covered while shaking. This resulted in a smaller headspace than when using 4 mL vials and 250 µL cell suspension. However, the oxygen measurements showed that there was no lack of oxygen during the reaction. So, with usually even more headspace it can be concluded that oxygen is not a limiting factor.

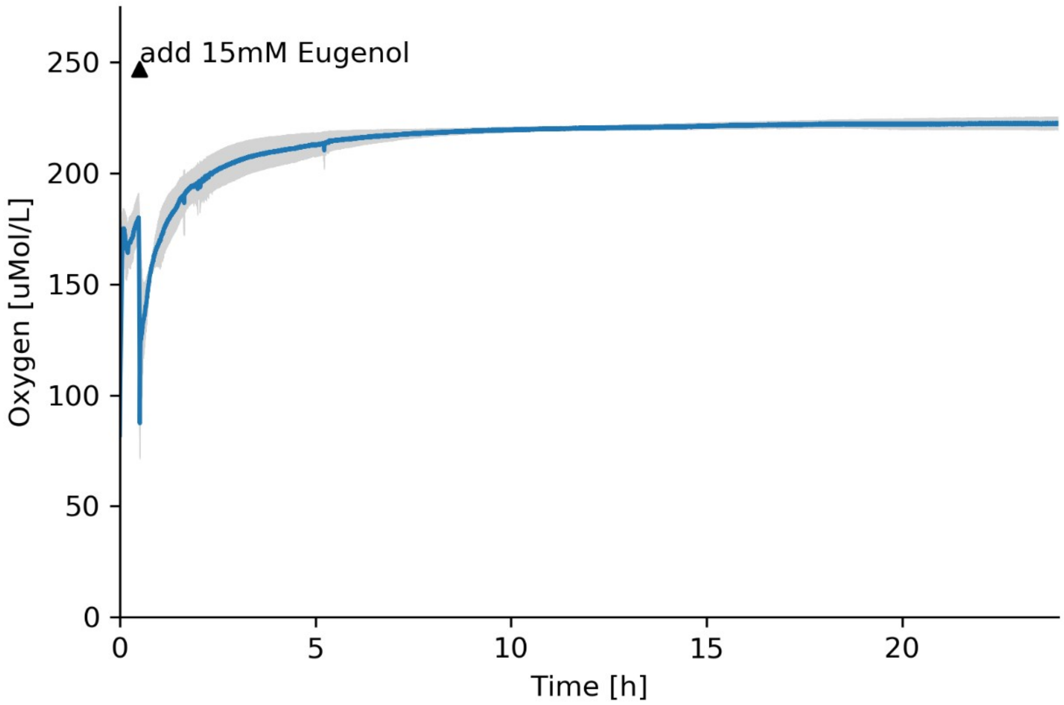

*Figure S7: Oxygen measurements during the cascade reaction with MapADO and ScEUGO using 15 mM **3a**.*

1.8 Kinetic data of selected alkene cleaving dioxygenases with **1b**

Selected enzymes of the pre-screening were purified, and their kinetic parameters were determined. The respective kinetic data are listed in Table S1. The purification of the enzyme *Ts*ADO was unfortunately unsuccessful therefore it is not included in the table.

*Table S1: Comparison of kinetic parameters of selected ADOs with **1b**.*

name	K_m [µM]	k_{cat} [s^{-1}]
ADO	2240 ± 236	67 ± 0.2
*Map*ADO	118 ± 12.7	238 ± 2.2
*Asp*ADO	1246 ± 627	40 ± 10.9
*Vs*ADO	393 ± 113.8	224 ± 23.3
*Pa*ADO	1043 ± 599.2	61 ± 17.8

2. Strains & Plasmids

In this work, the same bacterial strains were used as described in Schober *et al.*[2]: *E.coli* TOP 10 F' and *E.coli* TOP 10 were used for plasmid propagation, whereas *E.coli* BL21(DE3) was used for protein expression. The used plasmids are listed in Table S2 and Table S3.

Table S2: Used plasmids and vectors.

Plasmid	resistance	Usage	Ref
pET-21a	Ampicillin	Vector of origin of ADO (*Thermothelomyces thermophilus*)	[2]
pET-28a	Kanamycin	Protein expression in *E. coli* BL21 (DE3)	[2]
pET-16bp	Ampicillin	Protein expression in *E. coli* BL21 (DE3) for cascade reaction	This study

Table S3: Overview of all plasmids.

#	Name	Tag	Organism of origin	Accession No	Ref.
**pET-21**					
1	pET-21-ADO	N-terminal His tag	*Thermothelomyces thermophilus*	XP_003665585.1	[2]
**pET-28**					
2	pET-28-PsADO	N-terminal His tag	*Picea sitchensis*	ABR17001.1	This study
3	pET-28-TvADO	N-terminal His tag	*Talaromyces verruculosus*	KUL88876.1	This study
4	pET-28-TsADO	N-terminal His tag	*Talaromyces stipitatus*	XP_002483504.1	This study
5	pET-28-MapADO	N-terminal His tag	*Moesziomyces aphidis*	ETS60306.1	[2] and this study
6	pET-28-AspADO	N-terminal His tag	*Altererythrobacter* sp.	OJU58670.1	This study
7	pET-28-PaADO	N-terminal His tag	*Podospora anserina*	XP_001905181.1	This study
8	pET-28-MtADO	N-terminal His tag	*Minwuia thermotolerans*	WP_109794489.1	This study
9	pET-28-TtADO	N-terminal His tag	*Thermocatellispora tengchongensis*	WP_185048654.1	This study
10	pET-28-SlADO	N-terminal His tag	*Staphylotrichum longicolle*	KAG7284374.1	This study
11	pET-28-VsADO	N-terminal His tag	*Cytospora chrysosperma/*<br>*Valsa sordida*	ROV90719.1	This study
12	pET-28-CpADO	N-terminal His tag	*Coniochaeta pulveracea*	RKU43285.1	This study
**pET-16bp**					

13	pET-16bp-ScEUGO	N-terminal His tag	*Streptomyces cavernae*	WP_128378015	[3]

All relevant primers for cloning and assembly of the new ADO variants into one of the vectors listed in Table S2, are listed in Table S3.

Table S4: List of used primers.

#	name	sequence	description
1	insert fwd	CTCACTATAGGGGAATTGTG	Amplification of DNA insert
2	insert rev	CCAACTCAGCTTCCTTTC	

3. Gene Sequences

3.1 Sequence of *Ps*ADO

ATGGGGACTATGGCTGAAGATGACAGCAAGGGTTACAAGGCCGTAAATCCCCATCCCAA
AGGGGGCCTCGTCTCGTGGGTGGTGGACATGGTGGAGAAACTGGTGGTTGAAGCATCTAC
GTTGTACGGTTCGAGGAAGCCTATGCAATTTCTTTCGGGGAACTTCGCTCCAGTCTCGGA
AACTGCCCCCAAATCGCACTTGCCTGTTGTTGGGCATCTTCCCAGTTGCTTGGATGGGGAG
TTCGTGCGCGTCGGTCCCAATCCGAAATTCGCACCGGTAGCTGGCTATCACTGGTTTGATG
GAGATGGAATGATCCATGGTCTCAGAATTAAAGATGGTAAAGCCACTTATGTTTCACGTT
ATGTGAAGACATCACGCTTGAAACAAGAGGAATACTTTGGGAAAGCGAAATTCATGAAG
ATTGGAGACCTCAAAGGAATGTTTGGACTTTTCATTGTCCAAATACACATTTTACGCGAG
AAACTTGGTGTCCTAGACATGTCATATGGGAATGGAACAGCCAACACAGCCCTTATATAT
CATCATGGGAAGCTCCTTGCACTTCAAGAGGCAGATAAACCTTATGTGCTTAGAGTCCTT
GAAGATGGGGATTTGCAAACTCTTGGGCTAATGGATTATGATGATAAATTAGCACACTCC
TTCACTGCACATCCAAAGGTTGACCCTATTACAGGGGAGATGTTTACATTTGGTTATCAAC
ACACCCCACCCTATGTAACTTACCGGGTTGTTACAAAGGAGGGAATAATGCTTGATCCAG
TTCCTATAACACTTCCAAAACCTGTCATGATGCATGACTTTGCTATAACTGATAACTATGC
AATTTTCATGGATCTTCCTCTCTATTTTTCTCCAAAGGATATGGTAAAAGGTGGACTTATT
ATGTCTTACGATACTACAAAGAAAGCTCGTTTTGGTATATTGCCCCGATATGCCAAAGAT
GATCTTCAGATGCAATGGTTTGAACTGCCAAACTGCTACATCTTTCATAATGCGAATGCTT
GGGAGGAAGGTGATGAAGTTATCCTTTACACTTGTCGATTGCCTGACCTGGACTTGGAAA
TGGTCAGTGGAGCTGTCAAAGAAAATATGAAAAATTTTCAGAATGAACTATATGAAATGC
GATTCAATTTGAAAAGTGGAGAAGCCTCACAAAAGAAACTGTCCGAATCTGCAGTAGATT
TTCCAAGGATTAATGAGAAGTACATTGGCAGAAAGCAACGCTATGTATATGGTACTGTAC
TAGATTCAGTAACACAGGTTAAAGGACTGGTAAAATTTGATCTTCAAGCTGAACCTGAGA
TTGGAAAATCACAGATTGAAGTGGGAGGTAATGTGCTAGGGGTTTTCTGCTTTGGTCCTG
GAAGGTTTGGTTCTGAGGCAATTTTTGTACCCCGGGATTCTGGTCCGGACCTGGAAGAGG
ATGATGGTTACTTGATCTGTTTTGTGCATGATGAGAACACTGGGAAATCAGAGGTCAACG
TGTTTGATGCAAAAACAATGTCCCCTGAGCCAGTTGCTATAGTCTATCTTCCAACAAGGG
TACCATATGGATTTCATGCCTTCTTTGTCTCTGAGGAACAACTTCAAAACCAAGCATAG

3.2 Sequence of *Tv*ADO

ATGGCTGGTCACTTTCTTTCCCGCCTGGGGTCTCGCGCTGCCAATGGTTGGGGAAGCCTTT
ACAAGGAATGGCCGCAGATTGACACCCATTTCTCCTCTCAGCCTCCATGGGAAGGAGGTT
TCCACAGGCCCTCGCGGTTCGAAGGTGAAATCAACAACCTTGAGGTATATGGCGAGATCC
CCCACGAGATTGATGGCACCTTCTACCGCGTCATGCCTGAGCCGCAATTCCCTGCTTTCGC

AGATAAGAACGTGGTGTTCAACGGTGATGGTAACATCAGTGCGTTTCGAATTTCGAACGG
CCATGTGGACTTCAAGCAGCGCTATGTTCGCACAGAGAAATTCGTGCGCGAGGGTGAAGC
TCGTAGGGCTCTGATGGGCAAGTATCGCAACAGGTATACAGACTTGGTCCACTTTGAAGT
TCGGTCCACCGCGAACACCAATATCGTGTACTGGGATGGCAAGCTCCTTGCACTCAAGGA
AGACTCTCCTCCATATGCTATGGATCCGAACACGTTGGAAACGCTCGGCTTATACGACTTT
GGCGGTCAGCTCCCCGCATTGACCTTCACAGCTCATCCCAAGTTCGATCCGGCCACCCAT
GAAATGATATGTTTTGGATATGAGGCCAAAGGCGATGCTACCAATGACGTATGCTATTAC
ACCTTCGACAAGCAAGGTCGTTTGGTTGAGGAAGTCTGGGTCGATGCACCCGTCTGCGGC
ATGATCCATGACTTCGGAGTCACTGAGAACTATGTTCTCTTCCCCATAATTCCCTTACTCT
GCGATCTGGACCGACTCAAAGCTGGGGGAAATCACTGGCAGTGGGAAAACGATATCCCA
ATGTACATTGGAGTCCTGCCAAGGAGAGGAGCTAAAGGACGGACGTGAAGTGGTTCCG
AGCTCCTCATGGTTTTCCAGGCCACACAGCAAACGCATATGAAGATGCCGATGGTATTAT
CAATTTCCACACGACCTACTGTAAGGTCAACGCGTTCTTCTGGTGGCCCGACAAAAATGG
GGTTGCGCCAAAGCCAGACGAACTTGAGCCACGGTTGTACAATTTCAAGATAGATTACCA
CAGCAAAAACCTGGACCTCGGAGAGCCCGAGACCCTCCTGTCCACTGATAACTGTGAATT
CCCCAGAATTGATGACCGAATAGCCTTAGCGCGCCATTCCTGGGTTTTTATCAACGTCCTG
GACGTGGATGCTGGGACAGATCTGCCATTCGTGGCAACCCGCATGGGTGGTTCCCCTCCG
TTCAACACTATTGCTGCTTTCAATGTCAAGACCAAGGAATATAAGAAGTACTTCACAGGT
CCCCGTAAAACTACCCAAGAATGTGTCTTTGTGCCGCGGAATGCAGATGCCCCTGAGGGT
GATGGATTTGTTATTGCACTGTTGAATAATTACGAGGACATGGTGAGTGAGCTTGTGATC
TTGGATACTAGGGACTTTTCCAAGGAGGTGGCGCTGGTAAGGTTGCCTTTTACTCTACGTG
CCGGCCTGCACGGTAACTGGGTAGACAGCAGTGATGCAGATGGGCATCCGGGGACACTG
AAGGCTTTGTGA

3.3 Sequence of *Ts*ADO

ATGACTGAATACAAATCGGCGTTTCCCGATCTCCCCCAGTTTTCTGGGTTCATGAGACCCT
GTCGGCTCGAAGGCGAGGCATTCAACTTGGAAGTTCTGGGACAAATTCCCCAGGAAATCG
AGGGAACATTTTTTAGGGTTATGCCTGATCCCCAGACTCCCCCATTTATTGAAAATGACAC
GTGGTTCAACGGCGATGGCAACGTTTCTGCCTTTCGCGTCAAGGACGGCAAGGTGTCTTT
CAAACAGCGCTATGTGAGAACAGAAAAGTTTATACGAGAACGAGAGTCTCAACGAGCGC
TACTCGGCAAGTACCGCAACAAGTTCACAGACGCCGTAGAGTTCAGAATTCGCTCTACCG
CCAACACGAATATTGTTCATTTCAATGGTCAACTGCTAGCATTGAAGGAGGATTCGCCCC
CCTACGCGATGGACCCGATAACGCTGGAAACAAAGGGTCTTTACGATTTTGACGGGCAGT
TGCCAAGTCTGACATTTACAGCCCATCCAAAATTCGACCCGAAGACAGGTGAAATGGTGT
GTTTCGGATACGAAGCTAAGGGTGATGGCACACCGGATGTTTGTTATTACAACGTGACCG
CTGACGGCAAGTTCACCGAGACTGTTTGGCTGGTTGCTCCTGTTGTAGCGATGATTCACG
ATTTCGCCGTGACAGAGAACTGGGTTATATTCCCAATCATCCCCCAAGTCTGCGATCTGG
AGAGGATGATGCAGGGTGGGGAGCATTGGCAGTGGAGCTCAACGACACCAATGTATCTC
GGAGTGATCCCTCGTCATGGCGCCCAATCCACAGATGTGAAGTGGTTCCAATACAAGAAT
TCATTCCCCGGCCACACTGCGAATGCCTACGAAGACAAGAATGGCATCCTCGTTATCGAC
CTTGGCCTGAGCGAGAAGAACCAGTTTTTCTGGTGGCCAGATGCAGAAGGCAACGCTCCC
GAGCCCAGCTCAATCCATGGAGAGCTAGTGCGCTTCACCATAGATCCGCAGTCAGAGGAT
CTGAACCTGAGCGAACCCAAAGTTCTCCAGGCAGATAACTCCGAGTTCTATCGGATAGAT
GACCGATTCCTCATGCGGCCTTACCGGCATTGCTTTTTTGACATGATGGACCCAAAACTAG
GCACTGATTTCGAGCGTATCGCACCAAATATTGGGGGTGGTTATCCCCTTTACAACTCTCT
TGGCCACTTTGATAACCTCACGGGCAAAACAGAGGTATATTTCCCTGGGAAGGCACACAT
GGTCCAGGAGCCTGTTTTTATACCCCGACGCAATTCAACTGTCGAGGGTGATGGGTATAT
CATGGTACTAGTAAACAATTATGAGACAATGTCGAGTGAGCTGCACCTGTTGGACACGTC
TGATTTCACAAAACTGCAAGCAAAAATCCTGATTCCTGTGCGCTTGCGACACGGATTACA
CGGCAGTTGGGTTGATGGAAAAGAGCTTCCATTGCCTTCGCAGGCTGAATAA

3.4 Sequence of *Map*ADO

ATGGCTCCTACTGCAACGCAGGAGCCAGTGCCTGTGCCAGTCACTTCAAAAGCAGCTCCG
TCGCATGGATATGTCCATCCGACTGATATTCTGCCCTCGGGATGGCCCACCGCTACTGACT
TATCCGGCGGAGCACAGCCACGTCGTTTTGAGGGTACGATTTTTGACGTGATGACCCGTG
GCACAATTCCGAAGGAGCTTCACGGAACTTTTTACCGTATTATGCCTGACTATGCACAGC
CACCTACTTATTACAAGGGAGGAGAACTGAATGCTCCAATTGATGGAGACGGCACTGTGG
CCGCATTTCGCTTCAAGGATGGGAAGGTAGACTACCGTCAACGCTTCGTGGAAACGGATC
GTTTTAAGGTCGAGCGTCGCGCACGTAAATCTATGTACGGTCTGTACCGCAACCCGTACA
CGCACCACCCATGCGTACGCCAAACCGTAGAATCGACTGCCAACACCAACGTCGTCATGC
ACGCAGGCCGTTTTCTTGCCATGAAGGAAAATGGCAACGCATACGAAATGGACCCGCAcA
CTCTGAAGACCCTTGGTTATAACCCGTTCAATTTGCCATCTAAGACTATGACGGCACACCC
TAAGCAGTGCTCGGTTACCGGCAATTTGGTGGGCTTCGGATACGAGGCGAAGGGGTTGGC
AACCAAAGACGTGTATTATTTTGAGGTGGACCCCTCAGGAAAGGTAGTGCGCGACTTGTG
GTTAGAAGCCCCCTGGTGCGCGTTCATTCACGATTGCGCTCTTACGCCAAACTATCTGGTA
TTGATGTTGTGGCCGTTCGAGGCTAATCTTGAGCGCATGAAAGCCGGAGGACATCATTGG
GCCTACGACTACACGAAACCTATCACGTGGATCACGATCCCCCGTGGGGCTAAGTCTAAA
GATGAAGTAAAATACTGGCATTGGAAGAATGGGATGCCCATTCAcACCGCTAGCGGTTTC
GAAGACGAGCAAGGGCGCATTATCATTGATTCCTCCTTAGTGCACGGTAATGCCTTCCCG
TTTTTCCCTCCCGACAGTGATGAGCAGAAGAAAAAGCAGGAGGCTGATGGTACGCCTAA
AGCGCAATTTGTGCGTTGGACAATCGATCCCCGCAAAGATAACAATGAGCAACTTCCGGA
TCCCGAGGTGATCTTAGATACGCCTTCAGAATTCCCGCAAATTGATAATCGTTTTATGGGA
GTCGAGTATTCATCGGCATTTATTAACGTTTTCGTCCCTGACCGTTCGGACGGTAATAAGA
ACGTCTTTCAAGGCCTGAACGGCCTGGCTCACTATAAGCGTAAGGAAGGTACTACTGAGT
GGTATTATGCGGGAGATAATTGCTTGATCCAGGAACCAGTCTTTAGCCCTCGCTCGAAGG
ATGCGCCGGAAGGTGACGGGTTCGTGTTAGCTATCGTAGACCGTCTGGACTTAAACCGTT
CGGAAGTCGTTGTAATCGATACCCGCGACTTCACAAAAGCAGTCGCAGCGGTCCAACTTC
CTTTTGCTATTCGTTCGGGCATCCACGGGCAATGGATTCCTGGAGAGGTCACTCCAGATTT
TGAGACGAAGGGACTTGTCGATCTGCCGAAAGAGGAACATTGGGCGCCATTAAGTCAAT
CCCCATACGATCCCGACGCTTAA

3.5 Sequence of *Asp*ADO

ATGACAAAGCCTTTTCCCGACACGCCGACCTTTGCCGGCTTCAACGCGCCTATCCGCCTG
GAAGGGGAGATCTTCGACCTGGAGGTCGTTGAAGGCGAGATACCCACGGCGTTGGACGG
AACATGGTATCGTTGCGGAGCGGACTTCTATTTTCCGCCGCTCCACGGCGACGATGTGTTC
GTCAATGGCGACGGCGTTGTAAGCGCATTCGAATTCCGCGACGGGCATGTCGATTTTCGC
ACGCGTTTCGTCCGCACCGAGAAATTCGTCCTCGAGCGAGCGGCCCGCAAGGCCCTGTTC
GGCGCCTATCGGAATCCGTTCTCGGACGATCCTTCTGTCGCCGGGAAGAACCGTGGAACC
GCCAACACCAACATCGTGTGGCATGGCGGGAGGCTGCTAGCGCTCAAGGAAGCGGCGCG
GCCCGTCGAAATCGATCCGGTTACCCTCGAGACGATCGGTGATTGGGATTTCGACGCGCG
CCTCACCAGCCTGACAATGACAGCGCACCCCAAGTTCGATCCCTCAATCGGCGAGATGCT
GTTCTACGGCTCGGCCGCGAAAGGGGAGACCACTCCGGATATCGTCTACTACGCCGCCGA
TGCGGACGGTCGGATCACCAAGGAGATATGGATCGATCCGCCTTACGCCAGCATGATGCA
CGATTTCGGAGTGACGAAGGATCACGTCATCTTCCTCGTCATGCCGACCACCAGCAGCCT
GGAGCGGATGCGCGAAGGCGGACCGATTTACGTATGGGACGAAGATCTCGAAACCTATC
TCGGCGTGATGTCACGACATGGCGACGGTTCGGATTTGCGCTGGTTCACCGGGCCGCCGC
GGTTCGTCTTCCATATTCTCAATTGTTTCAGCGAAGGCGCGCTGGTCCACATCGATTCGGC
AGTATCCGAAATTCAATCGTTTCCGTTTTTTCCGGACAAGAATGGCCGGCCGTTCGACCCC
GAAAGGGCCAAAGCCAGGATCACGCGCTGGACCTGCGACCTGACCAGCAATTCGAGCGG
ATTCGTGGAGCGAACGATCAATCCGCTCGGCTGCGAACTGATCCGCATCGACGACCGGAT
CGCCTTGTCCAACTATACCTATGGATTCATTCCCTTCCATGACGCCGGCGCGCGTCAGGCG
GGGGACGCGAACGCCGGCTTCAATTCCGTCGGGCGCCTGAATGTCCGGACGGGAGAAAT

GGAGGACAGCTTTTTGCCGGCCCGAACGCCAGCGTCCAGGAACCGCAGTTCGTGCCGCG
TAGTGCGCAAGCGCAAGAAGGTGACGGCTACTTGCTGGTGATCGTCAATCGGCTCGATCA
AAACCGCTGCGATCTGGTGATATTGGATGCGAATTCCTTGAGCGGAGCCCCTGTCGCCCG
GATCAAGCTCCCCGTGCGTACGCGTGCAATCCACGGTAACTGGGTCGCCCGGGCGGAGTT
GCCCTGA

3.6 Sequence of *Pa*ADO

ATGGCGCACATTTTTCATCTTGCACCCCACATAGACACGAGCCCCAACTATAGCAACAAC
GAACTCACTAGTCCTGAAAAGGTAGCATTCCCGAAGACCGATGCGTTCCGATCCATGAAT
GCCCCCAGTCGTTTTGAGGGTTCGGTGTTCAATCTTGAAGTAACTGGAACAATACCCCCT
GATATTAGCGGTACCTTCTACCGTGTTCAGCCTGATCACCGGTTCCCGCCGCTGTATGAGG
ATGACATTCATTTTAATGGGGATGGCTCTGTTACTGCCATTCGTATAGCTAATGGGCATGC
CGATTTCCAACAGAAATATGTGGAAACCGATCGGTATAAGCACGAAACCGCGGCTCGGA
GATCACTGTTTGGCAAGTATCGAAACCCTTATACCGATAACGAATCCGTCAAAGGTGTAA
TTCGAACCGCATCAAATACAAACATTGTGTTTTGGCGTGGCATGCTGCTTGCAATGAAAG
AGGATGGACCTCCTTTTGCTATGGACCCTGATACGCTGGAAACCCTTGGGCGTTATGATTT
CGAGGGTCAAATTCTGAGTCCAACCTTTTGCGCTCATCCAAAATTCGACCCCGATACAGG
CGAAATGGTCTGCTTTGCGTACGAATGCGGGGGTGACGGTGCTGACTGCGGAGTCGATGT
GGCCGTCTGGACGGTAGACAAAGACGGTAAAAAAACACAGGAGGGCTGGTACAAAGCTC
CGTTTGCTGGCATGATTCATGATGCTGGACTGACTGAAAATTATCTGGTGCTTGCACTGAC
ACCGATTAAAATGAATCTGGAACGTCTGAAGCAGGGTAAGAATAAGTTTGCATGGGATC
CAGAGGAAGATCAATGGTATGCGGTTGTGCCGCGGCGGGGGACGGGCGATCCAGGTGAA
ATTACGTGGTTCCGGGGTGATAATGCTTTTCATGGCCATGTGGCTGGCGCGTATGAATTA
GCATCCGGAGAAATTGTATTCGATTTAACAGTAGCAGATGGCAATGTTTTCTTTTTTTTCC
CTCCGGATAAAAACGTAACTCCGTCTGCAGACAGTATAGCCAAGCGTAATAAACTCTCTT
CCCCTACAACACGATGGATATTTGATCCTAAAGCAAAGAAGTCCGCGATTCGCAAACCAG
AAGCTGGTGATTCGGACGTTTGGGTCGCAGACGAGCGAGTCAAACCCGCCGTTGTGTGGC
TTACTAATGGCGAGTTTTCCCGTATAGATGACCGTTACGTGACTAAACCGTATAGACATTT
CTGGCAGGCAGTTGTTGACCCCACCCGACCTTACGACTTTGCTAAATTAGGCCCGCCTGC
GGGCGGTCTGTTTAATTCTTTAGGTCACTATACTTGGGGGCCAGAACATTATCACACCGG
GAGCACCACATCATCAGTCACCAACGGTGAAACAAAGAAAGAAAAAGCAGGCTTGGAGG
ATGTCTACTTCCCCGGCCCCACAATGACCTTTCAGGAACCCACCTTTATACCGAAAGAAG
AAGGCGCCGAAGGTGAGGGCTACCTTATCGCACTGCTGAATCACCTTGACGTTCTGCGAA
ATGACGTCCTTATTTTCGACGCTCAGAACCTGTCTCAGGGTCCTCTCGCGGTAATTCATTT
ACCGTTAAAATTGAAACTTGGTCTGCATGGAAATTGGGTTGATAACAAAGATATCGAGGA
ATGGAAGGCGCGCCGTGAAGCCACAGGAGATGTGGGCCCGGTGAAGGTCGCCAGCGAAC
CACTGCCTTGGCAAAAAAAATTTCAGTCCGGTCAAGCAAACGGTCAAGAACGCTCATTCT
AA

3.7 Sequence of *Mt*ADO

ATGACGGCCCCGTTTCCGGATCATCCGCAGCTGAGTGGTAATTATGCACCCTTACTCATG
GAAGCTGACGCTCCGGATCTGGTGGTGCGAGGGGCAATACCCCCAGAATTAGATGGAAC
GTTGTTTCGCATTGGACCGAACCCTCAATATCCGCCTCGGGACCGCCAGCATCATTGGTTT
GCGGGCGACGGGATGGTCCATGCTTTTCGGATACGCGATGGCCGGGTTGGTTGGTCCAAT
CGCTGGGTACGTACTCCGAAGTTTGAACTTGAACGTGCGGCCGGTGAGGCATTGTTCGGA
ACTTTCGGTAATCCTCTGACATCAGCGGACGAGGCTCGTGGCAAAGATTCAGGGCTTGCA
AATACGAACATTGTTTGGCATGGAGGGCGTCTCCTGGCACTGGAAGAGGCGCATCCCCCA
TTTGAAATGAATCCAGCGGATCTGGCATCAAAAGGGTATTTTGATTTTGACGGTGGTCTG
ACCGGAGGACGTATGACCGCGCATCCAAAAATCGATCCCGCCACAGGAGAAATGCTGTT
CTTTGGGTACTCAGTTGGGGGGTACTTTACGGACCGCCTGTCGTATCATGTGGTCGATGCC
GGAGGTAACGTTACACAGGCGGCGGCTTTTGATGCCCCATATGCGGCAATGATCCATGAT

TTCATTGTTACCGAGGGACATGTAGTTTTTCCGGTGTTGCCGCTGACGGGTAGTCTGGAAC
GTGCTATGGCAGGACGCCCTCCGTTTGCATGGGAGCCGGATAAACCAGGTTGGTTGGGTG
TGTTGAAACGTGGTCAACACCCCGAGATGACACACTGGATTGAAATCCCGAGTTGCTACG
TTTTTCATCCTATGAACGCATTCGAAAGAGAAGGCCGTATTGTCGCCGATGTCATGAAAT
ATGAAGCTGCTCCTCTCTTCCCGCATGCCGACGGCACCCCCGGAGATCCTGCCAAGGCAA
CCGCTAGACTTGTACGCTGGACGATAGATCCTGCTCGTGATGAAGTCAAAGAGCAGGTGT
TGGATGGCCGACCCGGGGAGTTTCCCCGGTTTGATGAGCGTCGTGCAGGCCTGGGCTATC
GTCATGGCTGGTTTGCAGCAGTTGAACAACGTCGGAAAGGCGATCGCGGCAATTGGACA
GAAATTGTCCATGCCGATCTCGAATCAGATCGTCGCACAGTGTTTTCTGATGGCGAAGCT
CATCATGTGAGTGAGCCAGTTTTTGTACCTAGAGCACCCGATGCGGCAGAGGGTGATGGT
TGGCTGCTTTTCAATGTTTACAGAGCCGAACAAAACCGCTCTGATCTTGTCATCCTTAATG
CGCTTGACGTTGATGGAGAGCCCGTTGCCGTCGCCGAGATACCTTGTAGAGTTCCCAATG
GCTTTCATGGTAATTGGGCCCCGGGCGTGTAA

3.8 Sequence of *Tt*ADO

ATGTCAGACGGCAAGAACACAGGCAACCCGGTTTTCCGCGGTGCGTTCGCGCCGGCAAC
GGAAGAAATAACCGCCTTTGACCTGCCTGTAACCGGCGCTGTTCCTGCGGAGCTGAATGG
CCGCTTCCTTAGAAATGGCCCGAATCCCATCGATCTGGACGCGCCTAATCCGCACCTGTTT
GTAGGTGAGGGAATGGTACACGGTGTCCGTCTGCGGGACGGTCGGGCTGAATGGTATCG
GAATCGTTGGGTGCGTAGTGACGCTGTAGCGGAAGCTTTAGGGGAGGAACCACGCCCGG
GGCCGCGCTTCGACGGACTTGACTTTGCGGCGAATACTCACGTAATAGGTCATGCGGGTA
GAACATTGGCTCTGGTTGAGGCTGGACTTCCGGTATATGAACTTAGTGAAGAGCTTGAAA
CGTTGGGTCGGTGTGACTTCGGCGGTACCCTTCCTGGTGGATTTGCAGCGCATAGTAAGG
TAGATCCACGGACTGGAGAATTACATGCGGTCGCCTATTACTTTGGCTGGCAGGACAGAA
TCCAACATCTGGTTGTATCCCCTGACGGTAAGGTCGTACGTGCTGAGGACGTACCGGTAA
GTGATGGGCCGATGGTTCATGACTTTGCTCTGACAGAAAACCATGTAGTACTGTATGATC
TGCCCGTGACCTTTTCTATGGCCGCAGCGGAGGCCGGTGTGCGTTTGCCTTATGCGTGGA
ACGACGCTCATCCGGCCCGGGTTGGTTTGCTGCCGCGTCATGGTGGGGCAGCCGGTGTTC
GTTGGTATGAAGTGCCGCCATGTTTTGTATTTCACACCCTCAATGCTTACGAGGATGGTGA
AAAGGTGGTAGTCGACCTGGTGGCCTACGATCGCTTATTCGTGGAGGCCCGCTTAGACTT
AGGCGCACCACCTCGGCTCGAACGGTGGACTGTGGACCCGGCAGCTGGGCAAGTTCGTC
GTACTGTATTAGACGATCGTATGCAGGAATTCCCGCGGATCGATGACCGCCTGACGTCTC
GGCCGCATCGCTACGGATACACAGTTCTGACGGAAGCTTTTGCCGACTTCCTCCCTCGAC
GGGCGGAAGATCTTCAGGAACTGAGCGATCCGGCATTTGCTAATGCTCTGCTGAAGCACG
ACCTGTCTACGGGTGCCGCAGAGGCGCACGTATTCCCGCGTGGTTCTTATGCAGGGGAAC
CGGTGTTCGCTCCTAGCCCCAGCGGGGAAGCGGAGGATGACGGCTATATTATGACCTATG
TTAACAACCCGGAACGTGGGGCCGCAGACCTTGTCATATTAAGTGCGCAGGATTTCGCGG
GTCCACCAGTCGCCACGGTACACTTGCCGGCGCGTATCCCGTTAGGGTTTCATGGTAATTT
TGTACCGGACCCGGCATAA

3.9 Sequence of *Sl*ADO

ATGGACGAGTCGTCACGCCTGGAGGGGGATGTCTTTGACTTAGAAGTGACAGGTACACTG
CCGGCAGATATCAATGGAACGTTTTTTCGGGTTCAGCCTGATCATCGCTTCCCTCCACTGT
ATGAGGATGATATTCATTTTAACGGCGACGGCTCTGTAACAGCGATTCGCATCGCTGATG
GCCATGCCGACTTCAAGCAACGGTACGTGCGAACCGAGAGATATCAGGCCGAGACCCGG
GCACGGAAGTCCCTCTTCGGCAGATATCGAAATCCTTGGACGGACAACGAATCTATTCTT
AGTCCTACATTTACGGCTCATCCCAAGGTAGATCCAGAAACGGGAGAGATGGTTTGCTTT
GCATATGAAGCAGGTGGTGATGGAGCCGATTGTAGCGTAGACGTCGCAGTGTGGACAGT
GGATGCGGATGGCAAGAAGATCGAAGAGTGTTGGTATAAGGCCCCTTTTGCAGGTATGAT
ACACGACTGCGGTATATCCGAGAACTACGTCGTACTGGCCCTGACCCCTATCAAAATGGA
TTTTGAACGTATGAAGCGTGGTGGTAATAAATTTGCGTGGGATCCTAACGAAGACCAGTG

GTATGGTGTGGTACCTCGCCGAGGGGCAAAATCAGAGGACATCATTTGGTTTCGAGCAGA
CAACGGGTTCCACGGGCATGTGGCTGGGTGTTATGAACTGCCTTCTGGTGAAATCGTGTT
TGACTTGACTGTAGCAGATGGTAATGTTTTCTTTTTCTTCCCTCCGGATGATAATATCACG
CCGCCGGATGGAGTTGCCAAGCGCAATCGACTGTCTAGCCCGACAGTACGTTGGATATTT
GATCCTAAGGCTAAAAAAAGTGCTATTCGCACTCCTGCTGCCGGGGATGCGGACGTGTGG
GTCGCAGACGAACGTTCGAAGCCAGTTGTGACATGGCTGACAAACGGTGAATTTTCACGT
ATCGATGATCGGGTGACTACTAAGCCGTACCGGCATTTTTGGCAAGCAGTTGTTGATCCA
ACGCGACCTTACGATTTCGATAAATGCGGCCCGCCAGCTGGGGGCTTATTTAATTGCCTG
GGTCATTACACATGGTCTGAAGAACATTTTCACACTGATAAGCCTGCCCAGAATGGAACC
AACGGCAAGAGCGGCGAAGAGGGCAAGTTCGGTCTGGAAGACATGTATTTTCCGGGACC
CACTATGACCTTTCAGGAGCCAACGTTCATTCCTCGGGAGGGTGGAGCTGAAGGCGAAGG
TTATCTGATTGCCCTGCTGAACCACCTGGATCAGCTGCGTAATGACGTAGTTATTTTTGAT
GCACAGAACCTTGCAAAAGGCCCTTTAGCGACCGTTCACCTTCCGTTGAAACTCAAGCTG
GGTCTGCATGGCAATTGGGTGGATAATCGCGATATCGAGGCTTGGCAGCGGCGCAGAGG
AACAAATGGTGATGTGGGACCAGTGCAGGTAGCGACAGAACCCCTGCCGTGGCAGAAAA
AGTTGGCCGAGCAAAATGGGACTGGTGCCTAG

3.10 Sequence of *Vs*ADO

ATGGCACACATCTTCGACATAGCACCAGCTGTGAAAGGGTATGCCAAGGGTGTTTTGGCA
AGACCGGGTGAGGCCACTACATTCCCTGACACGGATGCCTTTCGGGGTGTGAACAAGCCA
ACGCGGTTTGAAGCGGATGTCTTCGATCTGGAAGTCACAGGGACCATTCCGAAGGAGATA
GATGGAACGTTCTACCGGATTCAGCCCGATCATCGTTTCCCTCCGCTGTTTGAAGACGATA
TACATTTCAACGGTGATGGATCTGTTACCGCTATCCGTATATCAGGTGGACACGCTGATTT
CAAACAACGGTATGTGCAGACAGATCGCTATAAAGCGGAAACGGCTGCCCGCCGTTCAC
TGTTTGGGCGTTATCGCAATCCGTGGACCGACAACGAGAGCGTGAAAGGGGTTATTCGTA
CGGCATCCAACACAAATATCACTTTTTGGCGCGGCATGCTCCTGGCTAGCAAGGAGGATG
GTCCGCCTTTTGCGATGGATCCGGTTACACTGGAGACCTTAGGCCGTTATGATTTTGAGGG
TCAGATTAAAGCCCCTACCTTTACGGCCCATCCTAAATTTGATCCGGATACAGGCGAAAT
GCTTTGTTTCGCATACGAGGCAGGGGGGGATGGTAACGACGGTAGCTGCGATGTTGTGGT
GTGGACGATAGACGCTGACGGTAAAAAAACCGAAGAGTGCCTGTATAAAGCACCGTATG
CAGGCATGATTCATGACATGGCTGTTTCAAAAAACTACGTGGTGCTGCCGCTCGCGCCGC
TGAAAGTGAACATTGATAGAATGAAAAAGGGGGGCGAAAAATTCGCATGGGATCCGAAA
GAAGACCAATGGTACGGTCTTGTGCCGCGTCGCAATGGAAAATCCGAGGACATTCGTTGG
TTTCGTGCCGATAGTGGTTTCCAGGGCCACATTGTTGGCTGTTATGAAAATGAGGAAGGT
AATGTAGTAGTGGACCTGAGTGTCGCTGACGGTAATGTTTTCTTTTGGTGGCCCCCAGAA
GATGGAGATGCACCTCCTGAAAAAACTACCAAACAGAACCAACTTAGTACTCCTACTACT
CGTTGGATTTTGGATCCAAAAGCTCCGACAAATACGCGCATTACACCATTTTACGTGTGG
CCTACAAATGGGGAGTTTAGTAGAATCGACGATCGATTTGTTACGAAAAAGTATAAACAC
TTCTGGCAGACTAAAGTCGATCCCACTCGCCCGTATGATCTGGAAAAGTGTGGGCCGCCG
GCAGGGGGACTGTTTAACTGTTTAGGCCACTACACCTGGGATATGGACAACCCGCAAGCT
AAAGGGGAAGAGGACGAATTCTTTTTTGGCCCTACGAGTACGTTCCAGGAACCTTCTTTT
ATACCGAAAGATGGCGGAGGTGAAGGAGAAGGCTATTTAGTAGCTTTAGTGAACCATTT
GGATGTCTTAAGAAATGATGTGGCAATTTTCGACGCTCTGCATGTCGCTAAGGGACCACT
GGCGGTGTTACATCTTCCTTTAAAACTGAAACTCGGTCTGCACGGTAATTTCGTTGACCAC
CGTGATATAAAGGCGTGGCAGCGGCGTCGTAATGCTAATGGCGATGTCGGACCTGTGCAG
GTCGCCACGGAACCTTTACCCTGGCAAAAAAGCTTTGCAAGCGGTGGCCAAAACGGTCTT
AATGGAGGCACCAATAAAAGCTAA

3.11 Sequence of *Cp*ADO

ATGGCCCACATATTTGACCTGGCACCTGAGGTCTCTTTACCGGACGAGCCGATTTACAAG
GATGGTAAACTGCATCGCCCTAATCATGTGCAGTTCCCGCAGACGCCTGTTTTTGCATCCA

TGAATAAACCCTCTCGTTTTGAGGGCACAATATTAAGCCTGGAGCATACTGGGATCATTC
CGCCAGAAATCAACGGGACATTTTTCGCGTCCAGCCGGACCATCGGTTCCCTCCGATGT
TCGAGGATGACATTCATTTCAATGGCGACGGCTCCGTTACAGCGATTCGCATCTTTGACG
GTAAGGTGGACTTTCGGCAGCGTTATGTCCACACGGAACGTTACAAAGCAGAGACAAAG
GCCCGTCGTAGTCTGTTTGGGCGTTACCGTAATCCGTGGACGGACAATGAGTCAGTGAAG
GGAGTCATCAGAACAGCCTCCAATACTAACGTCTTTTTCTGGCGTGGAATGTTACTGGCA
ACCAAGGAAGATGGGCCTCCGTATGCAATGGATCCCGTTACGTTGGAGACCTATGGTCGC
TATGATTTCGAAGGTCAGATACTGAGCCCGACCTTTACGGCGCATCCGAAATTTGATCCG
CGTACCGGTGAAATGGTTTGCTTTGCATACGAGACTGGCGGAGATGGCGCAGATTGTTCG
CGTGAGGTGATGGTATGGACACTCGACAAGGATGGGAAAAAAGTTTCGGAAAGATGGTT
TGAGGCTCCTTTTGCGGGCATGATTCACGATTGCGGGTTAAGTGAGAACTGGCTCGTTTTA
CCCCTTACGCCAATCAAAATGGATCTGGAACGTATGAAACGCGGTGGTAACAAATTTGCG
TGGGATCCGAAAGAAGATCAAGTTTATGGCTTGGTCCCGCGTCGTGGTGATGGTGAAGTT
AAATGGTTTCGGGGTGAAAACGCTTTTCATGGACATGTGGCAGGCTGTTATGAGAATGCT
CAAGGCCATGTGGTGATCGATTTAACGGTCGCCGATGGGAATGTTTTCTTCTGGTTTCCCC
CAGACGGTGAAGAACAGGGCCAATTCGCGAAAAGAAATAAACTGTCTAGTCCGACCCAC
CGTTGGATTTTAGATCCCTCTTTACCAAATAATGCTAGAATCACACCTGCATTAGTATGGC
CGACGAATGGTGAATTCTCACGGATCGACGATCGGTGGACCACGCGCAAGTATAAACATT
TTTGGTTGGCTAAAGTGGATCCTAGCCGGCCCTACGACTTTGCGAAGTGTGGTCCACCGG
CGGGTGGCTTGTTTAATTGCCTTGGCCATTATACATGGGACCTGGACAACGAGCTGGCAA
CAGGTCAGGAAGATGTTTATTTCGCCGGCCCGACCTGCACCTTTCAAGAGCCCACATTTA
TCCCTAAGGGGGATAAAGAGGGAGAAGGCTGGCTGATCGCGTTAGTCAATCATTTAGAT
GTGCTGCGGAATGATGTTGTAATACTCGATGCTCAAAATTTAGCCAAAGGCCCTGTTTGC
ACAATCCACCTTCCCTTAAAGTTGAAACTGGGACTGCACGGCAATTGGGTGGACTGGCGT
GATATCGAAGATTGGACAAAGCGTCGGCAGGAAGATGGCGAAGTGGGGCCTGTACAAGT
TGCCACGGAAATGCTTCCGTGGCAAAAAGCTTTTTGGGAGAAGGAAAAAGAGAAAAATG
GCAATGGCGTGGAGGGCCCGAATATCAATGGAACGAACGGCGCTAACGGGACGAATGGT
GTAAACGGCTCATCCCATTAA

3.12 Sequence of eugenol oxidase (*Sc*EUGO) from *Streptomyces cavernae*

ATGACGCGCACACTGCCGCCTGGCGTGAGTGATGAGGACTTCACCAGCGCGCTGACCGCA
TTTCGCGATGTTGTGGGTGACGAGTTTGTTCGCACGGATGAGGCTGAACTGGCCCGCTTTC
ACGATCCGTACCCGGTTGGAGATGCTGATGCTCATTTAGCCTCTGCGGTGATTAGCCCTCG
CGACACGGAACAAGTACAGGAAGTCGTGCGCATTGCAAACCGCTATGGCATTCCGCTTTC
GGTGATTTCAACTGGCCGGAATAATGGCTATGGCGGTAGTGCGCCGCGTTTAAGCGGCGC
GGTTGTTGTGAATACGGGCGAACGCATGAACCGCATTCTGGAAGTGGATGAGAAACTGG
GATACGCGCTGTTGGAACCTGGCGTGACATACTTCGATCTGCACGAATACCTTGAAGCCC
ATGCACCGTCGTTAATGATTGACTGCCCGGATCTGGGTTGGGGTTCGGTGGTTGGGAACG
CGTTAGATCGTGGGGCAGGCTATACCCCGTATGGGGATCACTTCATGTGGCAAACTGGTA
TGGAAGTAGTCCTTCCACAGGGTGATGTTATGCGTACTGGCATGGGCGCCTTACCGGGTA
GCACGACATGGCAGCTCATTCCGTATGGTTTTGGACCATACCCAGACGGCATGTTCACCC
AGTCCAACCTGGGTATTGTCACGAAAATGGGCATTGCACTCATGCAGAAACCGCCAGCGT
CCATGACCTATCAGATCACGTTTGAGAACGAAAGCGATCTGGAGCAGATCGTCGACATCA
TGCTGCCACTGCGTATCAATATGGCTCCGCTGCAGAATGTACCGGTTCTGCGCAACATCA
TCCTCGATGCCGCCGTAGTGTCTCAACGGGCCGATTGGTACGATGGGGATGGGCCTCTGC
CGCCCGAAGCGATCGAACGCATGAAGAAAGAGCTGGGCTTGGGTTACTGGAATTTCTAC
GGCACCCTGTATGGCCCACCGCAACTCATCGAAATGAACTACGGCATTATTAAGGACGCC
TTTGGCCAGATTCCTGGTTCACGCTTTCAGACCCATGAGGAACGTCACGATCGTGGAGCA
CATGTCTTGCAAGATCGCCACAAAATCAACAATGGTATCCCCTCCCTGTCTGAGATGAAA
CTTATGGACTGGATTCCCGGTGCAGGACATGTCGGTTTTAGCCCGATCAGTCCGCCGGTA
GGTCGTGACGCTATGAAACAGTTCCGCATGGTGCGTTCACGTGCGGACGAATATGCGAAG

GACTATGCAGCGCAGTTTGTGGTCGGGTTACGGGAAATGCACCATATTGCGCTGCTTCTG
TTTGATACCCAAGACGCGACAGCACGTAATGAAACCTTGGCCTTGACTCGTCTGCTGATT
GATGAAGCTGCTGCCGAAGGGTATGGCGAATATCGTACCCATAATGCCCTGATGGATCAA
GTTATGGGCACCTATAACTGGGGCGATGGCGCGCTGCTGAAATTCCATGAAGCGATCAAA
GACGCCCTCGACCCCAACGGTATTATTGCGCCTGGTAAATCGGGTGTGTGGCCAGCACGC
TATCGCGGGAAAGGATTGGCGGCCGCACTTAAGTTACGCGTGGATCCGGCTGCTAACAAA
GCCCGAAAGGAAGCTGAGTTGGCTGCTGCCACCGCTGAGCAATAA

4. Methods

4.1 Preparation of ADO plasmids

Materials from New England Biolabs were used for PCR reactions. Primers were ordered from IDT. PCR reaction fragments were purified using the Wizard® SV Gel and PCR-Clean-Up System kit. Assembled circular vectors were desalted and used for transformation of electrocompetent *E. coli* Top 10F'. Positive clones were selected on LB-Amp or LB-Kan agar, and plasmids were isolated using the Wizard® SV Miniprep DNA Purification System. DNA temples were codon optimized for *E. coli* and ordered from twist gene. The sequences were verified through Sanger Sequencing service (Microsynth).

The genes of the various ADOs were constructed by PCR amplification of templates ordered from Twist Bioscience. The PCR reaction of 25 µL contained 5 ng of DNA template, 0.48 µM primer pair (Table S), 200 µM dNTPs, and 0.02 U/µL Phusion High-Fidelity DNA polymerase in 1X Phusion HF Buffer or CG buffer containing 5 % DMSO. The PCR reactions were started with an initial denaturation at 98 °C for 60 seconds. Subsequently, 30 cycles of denaturation at 98 °C for 10 seconds, annealing at 60-70 °C for 30 seconds, and extension at 72 °C for 3 minutes were carried out, followed by a final extension at 72 °C for 10 minutes.

For the backbone, the plasmid of a pET-28a empty vector was isolated using the Wizard® SV Miniprep DNA Purification System, then digested with *Xba*I and *Hind*III using FastDigest Restriction Enzymes standard protocol. The digested DNA was then separated by gel electrophoresis with a 1 % agarose gel containing 5 µL ethidium bromide. The gel was run at 120 V for 100 to 120 min and the desired band was cut and purified using a Wizard® SV Gel and PCR-Clean-Up System kit.

PCR product and restriction digested pet-28a backbone were combined with Gibson assembly using its standard protocol.

E. coli TOP 10 F' competent cells were transformed with the Gibson assembly mix by electroporation. The transformed cells were spread on an LB agar plate containing 100 µg/mL Kanamycin and incubated at 37° C for 16 h. A single colony from each plate was grown and the plasmid DNA was extracted and verified by DNA sequencing. *E. coli* BL21(DE3) was transformed with the verified plasmid of each ADO variant to express protein according to the protocol described above.

4.2 Enzyme expression

4.2.1. General flask expression protocol for all ADOs

10 mL LB media with antibiotics (final concentration 100 µg/mL Ampicillin or 50 µg/mL Kanamycin) was inoculated with a single colony or from glycerol stock and grown as the overnight culture (ONC). ONCs were incubated at 37 °C/120 rpm for 16 h. Main cultures consisting of LB media with antibiotics (final concentration 100 µg/mL Ampicillin or 50 µg/mL Kanamycin) were inoculated with 2 % ONC. Main cultures were incubated at 37 °C/120 rpm. At an OD_{600} of 0.6 to 0.8 protein expression was induced by addition of 1 mM IPTG and 1 mM $FeCl_2$ supplementation. For expression, the cultures were

incubated at 20 °C/100 rpm for 16 to 20 h. Cells were harvested by centrifugation of the culture at 4 °C/4000 rpm for 30 min in an Eppendorf 5810 R centrifuge (≤ 50 mL volumes per vessel) or at 4 °C/5000 rpm for 30 min in an Avanti JXN-26 (rotor: JA-10 Fixed-Angle Rotor- 6 x 500 mL) (≤ 500 mL volumes per vessel). The pellets were washed twice with storage buffer (10 mM phosphate buffer pH 7.4) or the desired reaction buffer e.g., 100 mM TrisHCl (pH 9.5) or 20 mM HEPES (pH 8.0).

4.2.2. General flask expression protocol for ScEUGO

A colony was picked from a LB-agar plate or a cryostock and grown in 4 mL LB medium supplemented with the respective antibiotic (Ampicillin, stock concentration 100 mg/mL; working concentration 100 µg/mL). The culture tube was incubated at 37 °C at 170 rpm for 16-20 h.
For the main culture, 100 mL TB-autoinduction medium, 100 µL Ampicillin and 1 mL of the overnight culture were put into a baffled flask. The flask was shaken at 140 rpm at 37 °C until the OD_{590} reached 0.4-0.5. Then the flask was incubated in a shaker at 20 °C and 120 rpm overnight.
After OD measurement, the culture was transferred into a falcon tube (50 mL, PP tube, Greiner bio-one). After centrifugation (6000xg, 4 °C, 8 min, SIGMA 3K30 or 6K15 centrifuge), the supernatant was discarded. The pellet was resuspended in PBS buffer (10 mM pH 7.4) and centrifuged. This washing step was repeated twice. Finally, the pellet was resuspended in PBS buffer to the desired optical density.

4.3 Enzyme purification

Cells were harvested by centrifugation for 30 min at 5000 rpm in a precooled Avanti JXN-26 (4°C) (rotor: JA-10 Fixed-Angle Rotor- 6 x 500 mL) and washed twice with respective binding buffer or similar. The cell pellet was resuspended in binding buffer (25 mM phosphate buffer, 500 mM NaCl, 20 mM imidazole pH 7.4) and sonicated 3 times for 120 s at 70 % amplitude on ice. Cell fragments were removed by centrifugation for 30 min at 20000 rpm in a precooled Avanti JXN-26 (4 °C) (rotor: JA-25.50 Fixed-Angle Rotor- 8 x 50 mL). The supernatant was filtered through a syringe filter (0.45 µm) to remove residual particles before samples were loaded on a HisTrap FF 5 mL cartridge via sample pump (flow:5 mL/min). The column was washed with 10 CV binding buffer until no absorption was detected in the flowthrough, then the enzyme was eluted with 20 CV elution buffer (25 mM phosphate buffer, 500 mM NaCl, 500 mM imidazole (pH 7.4)) with a flow of 1 mL/min and a gradient to 100 % elution buffer over 100 min. Flow-through was collected in 1 mL fractions. Fractions containing the purified enzyme were pooled, the buffer was exchanged for storage buffer (10 mM phosphate buffer pH 7.4) and samples were concentrated using Vivaspin® Centrifugal Concentrators and stored at -20 °C.

4.4 Kinetics

Catalytic constants such as K_m and k_{cat} were determined for **1b**, **2b**, **3b** and **5b**. For the determination of kinetic parameters, the generation of products, **1c** and in case of **5b** to 4-hydroxybenzaldehyde (**4c**) and 3,5-dihydroxybenzaldehyde (**5c**) were monitored by UV absorption on plate reader or UHPLC.
1b and **3b** were measured on a plate reader to monitor initial rates of **1c** production. Here the reactions contained potassium phosphate buffer (10 mM, pH 7.4), substrate (**1b**: 0.04 mM to 1 mM; **3b**: 0.01 mM to 2.5 mM) and EtOH (2 % v/v). Reactions were prepared in triplicates. The final reaction volume was 100 µL. Reactions were incubated and measured at 30 °C.
5b and **2b** were measured on UHPLC with the same method for the analysis of the biotransformation since absorption spectra of substrate and products possess similar peaks. Here the reactions contained potassium phosphate buffer (10 mM, pH 7.4), substrate (**5b**: 0.04 mM to 1 mM; **2b**: 0.01 mM to 2 mM) and EtOH (2 % v/v). Reactions were prepared in triplicates. The final reaction volume was 50 µL. Reactions were incubated at 30 °C/800 rpm and were terminated by adding 150 µL of MeOH. The sample was vortexed for 5 minutes and centrifugated for 10 minutes at 15000 rpm to remove the enzyme and other solid materials prior to UHPLC analysis.

4.5 Buffer and media

***LB medium*:**[3] 4 g bacterial peptone, 2 g yeast extract and 4 g NaCl were dissolved in 400 mL deionized water and the solution was autoclaved.

***TB medium**:* For the TB-autoinduction medium, 4.8 g peptone and 9.6 g yeast extract were dissolved in 392 mL PPB buffer and autoclaved. Afterwards, 8 mL of 50x5052 were added to the TB-autoinduction medium.

***PPB buffer**:* For the PPB buffer 7.48 g K_2HPO_4 and 5.047 g KH_2PO_4 were dissolved in 800 mL deionized water and the solution was autoclaved.

50x5052: 50x5052 was prepared by dissolving 125 g glycerol, 12.5 g glucose and 50 g α-lactose in 500 mL deionized water. The solution was sterile-filtered.

***PBS buffer**:* For 10x PBS 80 g NaCl, 2 g KCl, 14.4 g Na_2HPO_4 and 2.4 g KH_2PO_4 were dissolved in 800 mL distilled water. The pH was adjusted to pH 7.4 and distilled water was added until 1 L was reached. The buffer was autoclaved.

Phosphate buffer: For 0.2 M phosphate buffer 4.64 g KH_2PO_4 and 28.9 g K_2HPO_4 were dissolved in 800 mL distilled water. The pH was adjusted to pH 7.4 and distilled water was added until 1 L was reached. The buffer was autoclaved.

***2x SDS PAGE sample buffer*:** 125 mM Tris-HCl (pH 6.8), 4 % (w/v) SDS, 20 % glycerol, 100 mM DTT, 0.02 % (w/v) bromophenol blue and 1.33 % β-mercaptoethanol were mixed.

SDS PAGE running buffer: Commercial ROTIPHORESE® 10x SDS-PAGE (ROTH) was used.

5. Analytics

5.1 SDS-PAGE

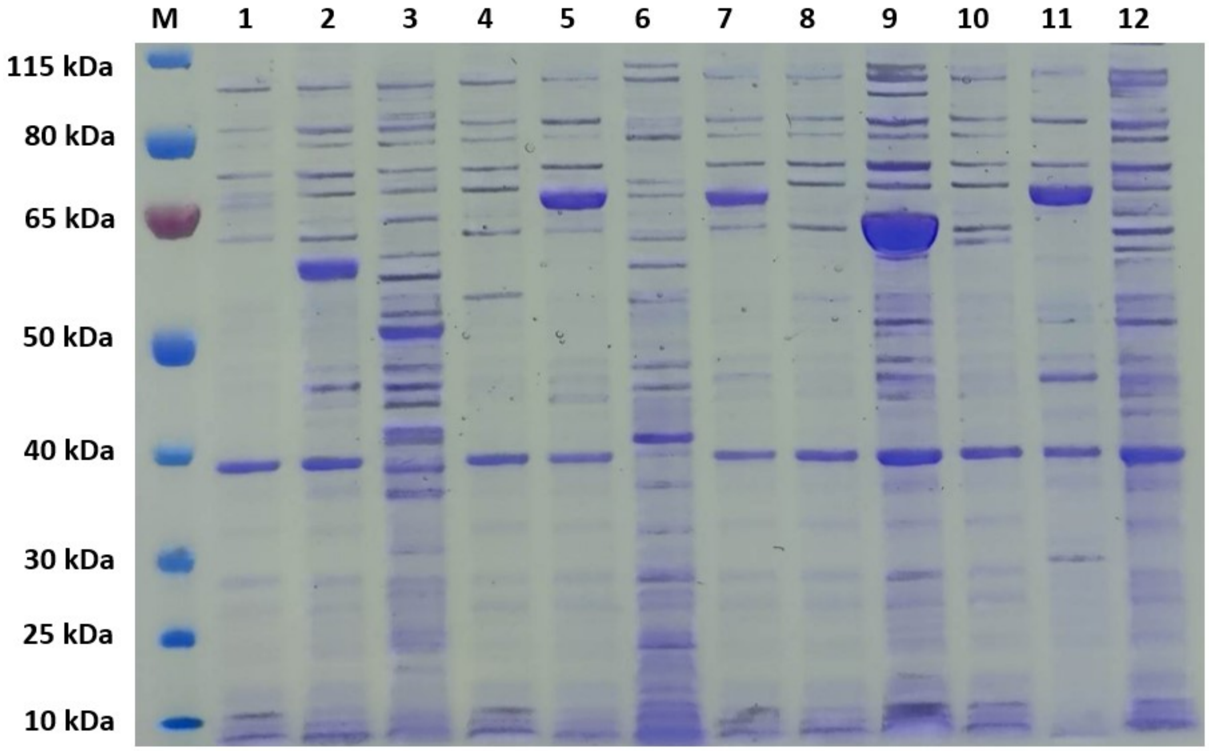

Figure S8: *SDS-PAGE with Coomassie blue staining of the soluble supernatant fraction from cell lysate of ADO variants: M, PageRuler™ Prestained Protein Ladder, 10 to 180 kDa; lane 1,* Cp*ADO from* Coniochaeta pulveracea *(69.6 kDa); lane 2,* Asp*ADO from* Altererythrobacter *sp. 66-12 (57.2 kDa); lane 3,* Ps*ADO from* Picea sitchensis *(64.2 kDa); lane 4,* Tt*ADO from*

Thermocatellispora tengchongensis *(55.3 kDa); lane 5,* Pa*ADO from* Podospora anserina *S mat+ (71.3 kDa); lane 6,* Ts*ADO from* Talaromyces stipitatus *ATCC 10500 (59.1 kDa); lane 7,* Vs*ADO from* Valsa sordida*(67.4 kDa); lane 8,* Mt*ADO from* Minwuia thermotolerans *(55.9 kDa); lane 9,* Map*ADO from* Moesziomyces aphidis *DSM 70725 (65.2 kDa); lane 10,* Sl*ADO from* Staphylotrichum longicolle *(60.5 kDa); lane 11, ADO from* Thermothelomyces thermophilus *(67.3 kDa); lane 12,* Tv*ADO from* Talaromyces verruculosus *(62.3 kDa).* All ADO were expressed in a pET-28 vector with an *N*-terminal His tag; All protein fractions were produced in *E. coli* BL21 (DE3) induced with 1 mM IPTG and 1 mM $FeCl_2$ added, expression at 20 °C/100 rpm for ~ 18 h.

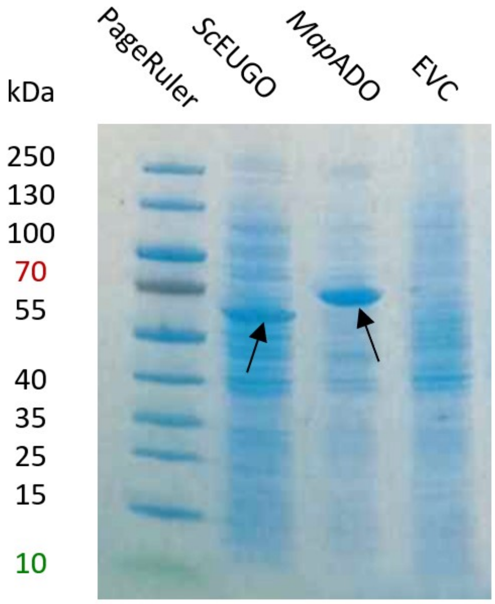

Figure S9: SDS-PAGE for expression control: The samples were denatured with a SDS-sample buffer at 95 °C for 10 min. Mini-PROTEAN TGX Gels BIO-RAD stain-free gels were used for SDS-PAGE, run at 200 V and stained with SimplyBlue™ SafeStain (InvitrogenTM). PageRuler (Fisher Scientific, Waltham, MA, USA) was used as a marker. The lanes from left to right show: PageRuler, ScEUGO, MapADO and empty vector control.

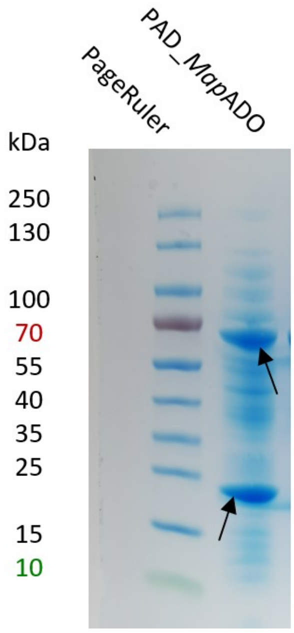

Figure S10: SDS-PAGE for expression control of E.coli BL21 pET-28_PAD_MapADO. 15 µL of whole cell sample were loaded onto a BioRad 4-18 % gel. PageRuler (Fisher Scientific, Waltham, MA, USA) was used as a marker. The expected bands are 65 kDa for MapADO and 19.2 kDa for PAD.

5.2 HPLC

Reactions were analysed by HPLC measurements. In the following section the retention times and calibration curves using Shimadzu Nexera HPLC device with SPD-M40 photodiode array detector are shown. The measurements for the eugenol cascade reaction were measured with the basic method, for all the

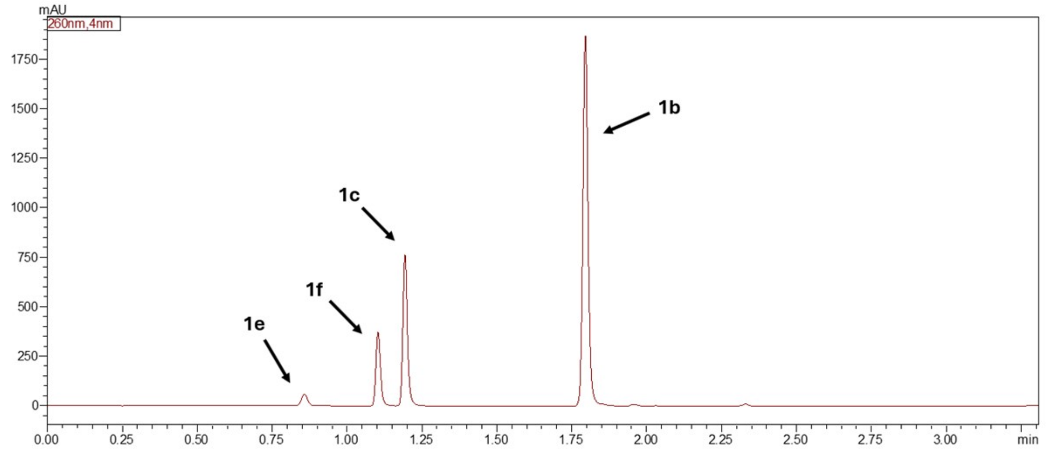

others the acidic method was used. For quantification the PDA peak areas were analysed.

*Figure S11: Standard mix of isoeugenol (**1b**), vanillin (**1c**), vanillyl alcohol (**1e**) and vanillic acid (**1f**).*

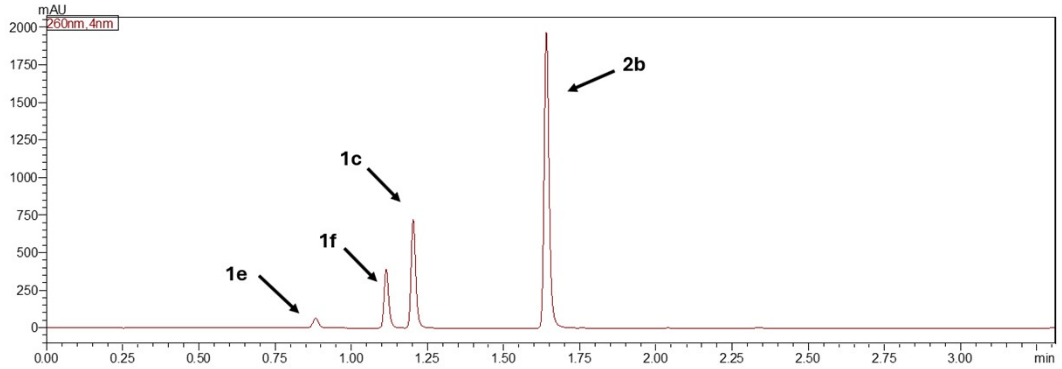

*Figure S12: Standard mix of 4-vinyl guaiacol (**2b**), vanillin (**1c**), vanillyl alcohol (**1e**) and vanillic acid (**1f**).*

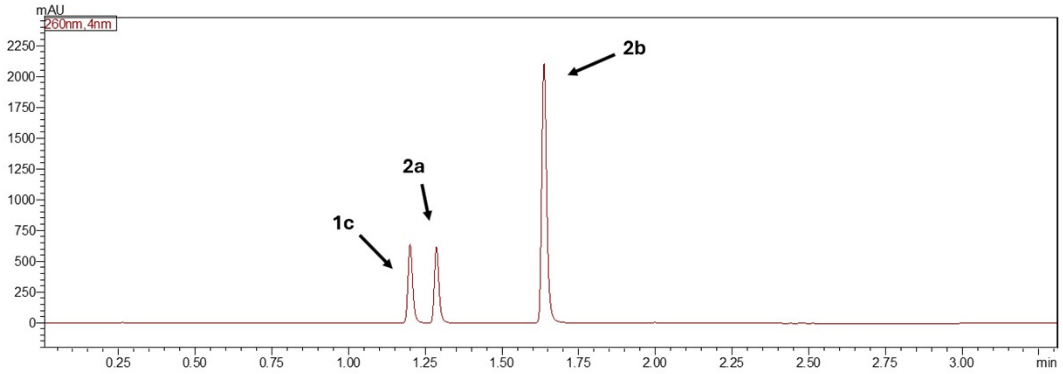

*Figure S13: Standard mix of ferulic acid (**2a**), 4-vinylguaiacol (**2b**) and vanillin (**1c**).*

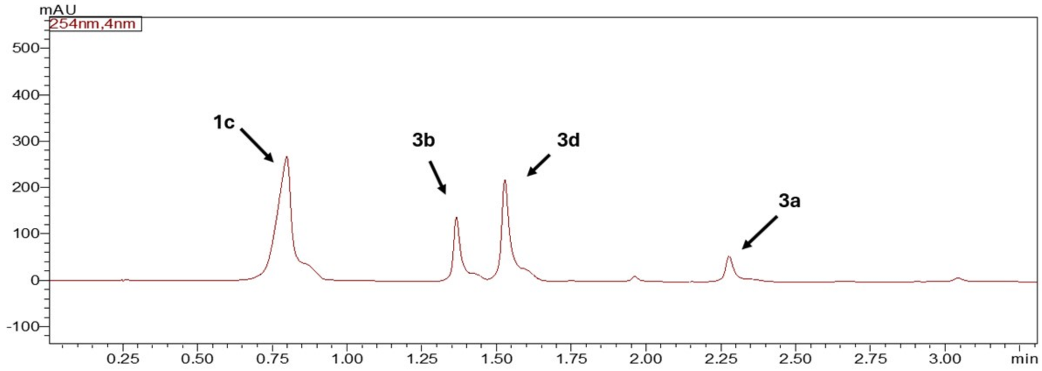

*Figure S14: Standard mix of eugenol (**3a**), coniferyl alcohol (**3b**), coniferyl aldehyde (**3d**) and vanillin (**1c**).*

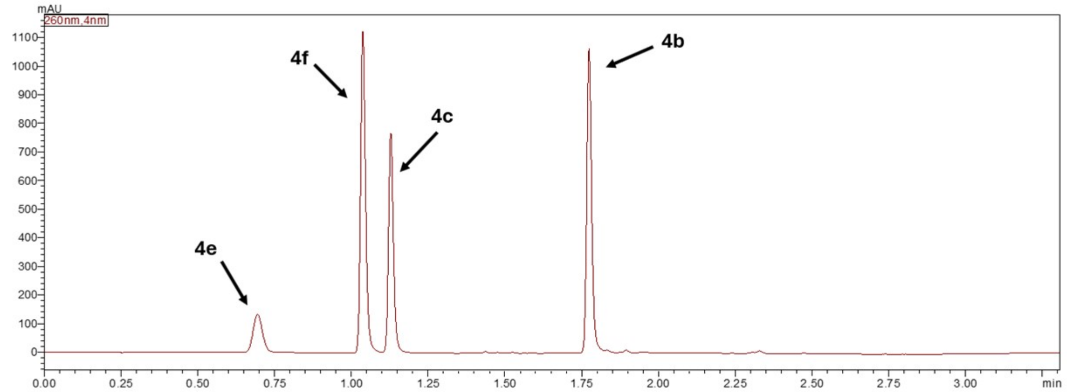

*Figure S15: Standard mix of hydroxyanethole (**4b**), 4-hydroxybenzaldehyde (**4c**), 4-hydroxybenzyl alcohol (**4e**) and 4-hydroxybenzoic acid (**4f**).*

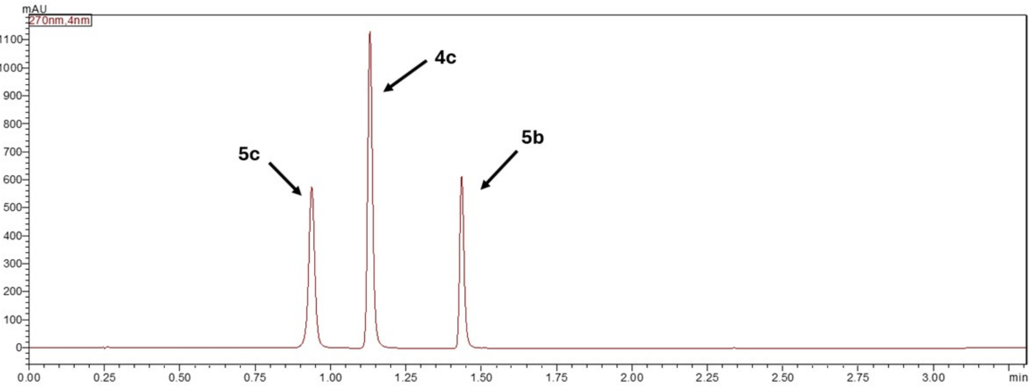

*Figure S16: Standard mix of resveratrol (**5b**), 4-hydroxybenzaldehyde (**4c**) and 3,5-dihydroxybenzaldehyde (**5c**).*

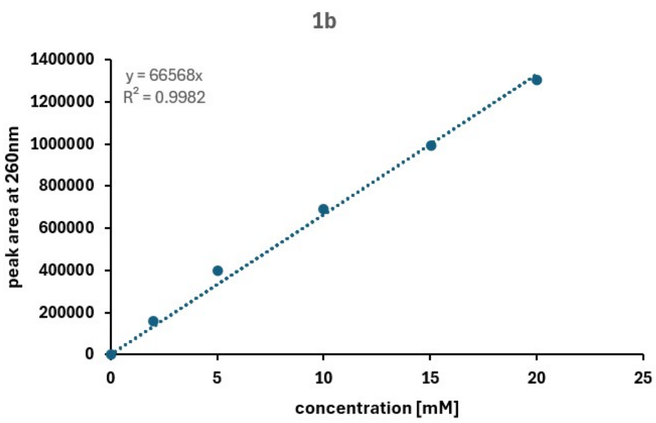

Figure S17: Calibration curve of isoeugenol (**1b**) using the acidic method.

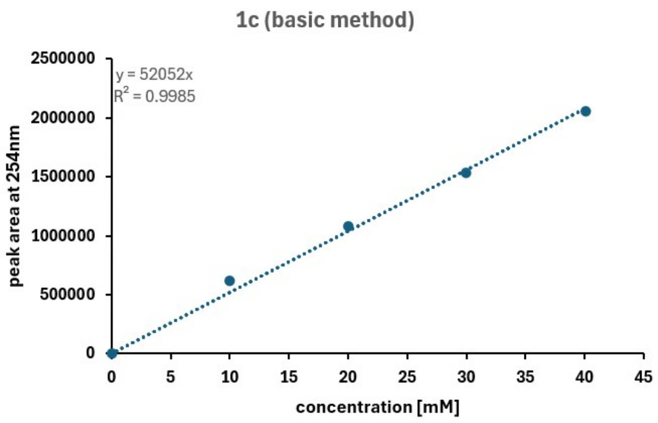

Figure S18: Calibration curve of vanillin (**1c**) using the basic method.

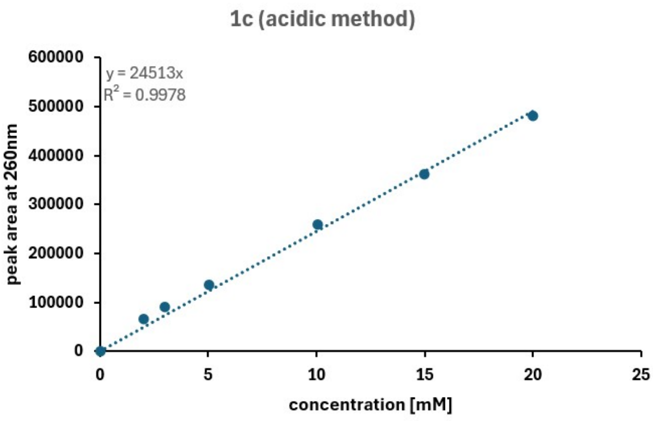

Figure S19: Calibration curve of vanillin (**1c**) using the acidic method.

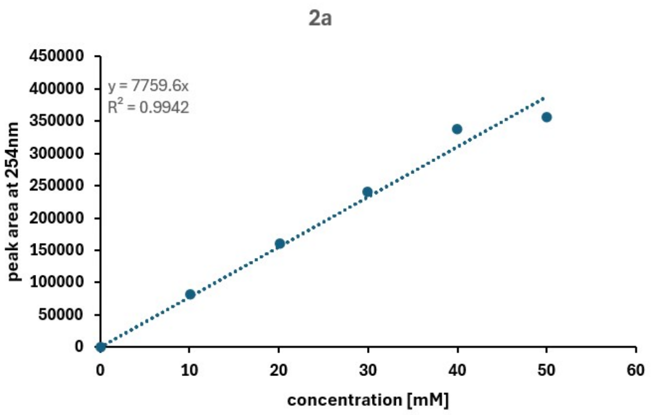

Figure S20: Calibration curve of ferulic acid (**2a**) using the acidic method.

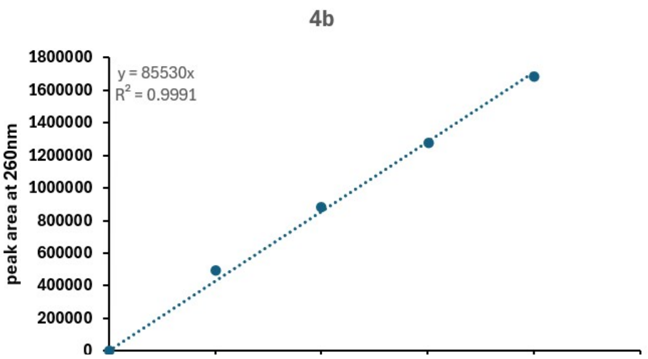

Figure S21: Calibration curve of hydroxyanethole (**4b**) using the acidic method.

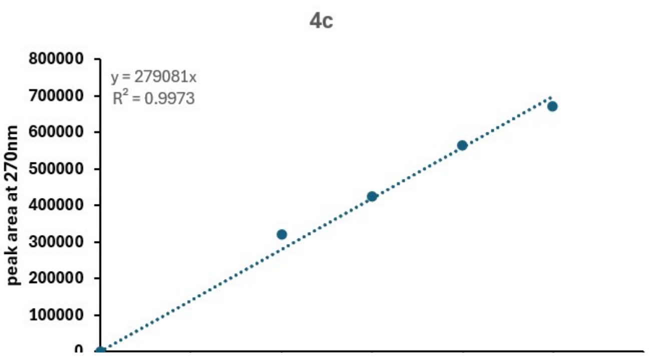

Figure S22: Calibration curve of 4-hydroxybenzaldehyde (**4c**) using the acidic method.

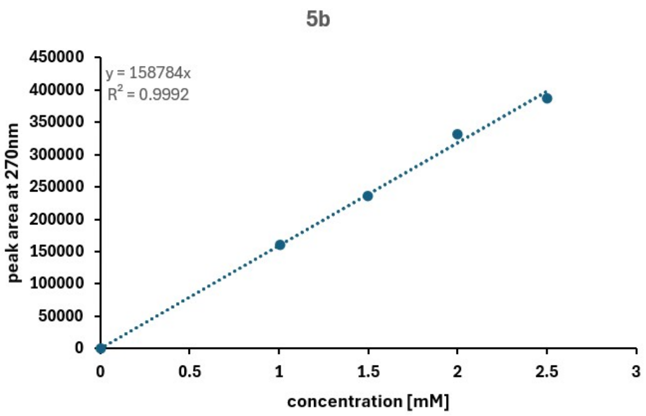

*Figure S23: Calibration curve of resveratrol (**5b**) using the acidic method.*

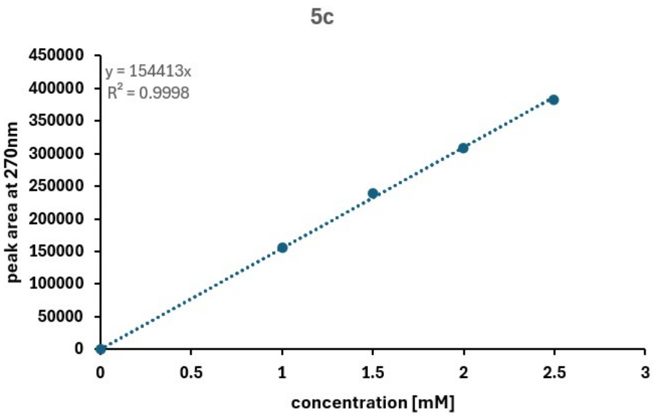

*Figure S24: Calibration curve of 3,5-dihydroxybenzaldehyde (**5c**) using the acidic method.*

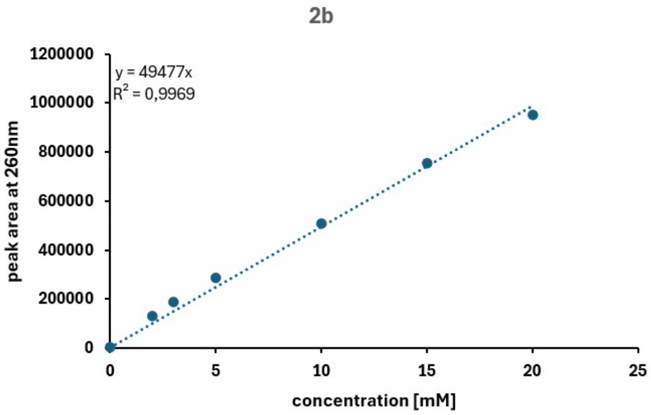

*Figure S25: Calibration curve of 4-vinylguaiacol (**2b**) using the acidic method.*

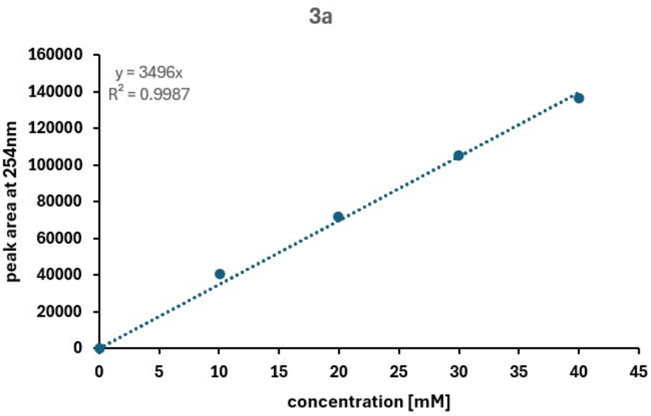

*Figure S26: Calibration curve of eugenol (**3a**) using the basic method.*

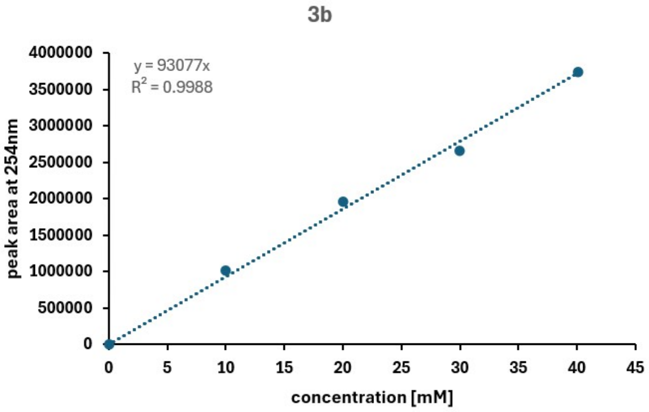

*Figure S27: Calibration curve of coniferyl alcohol (**3b**) using the basic method.*

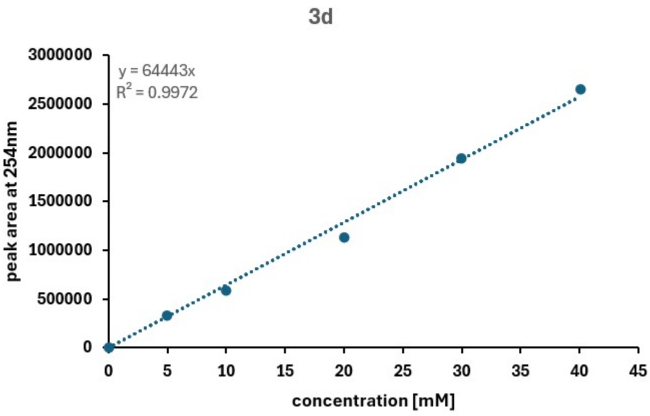

*Figure S28: Calibration curve of coniferyl aldehyde (**3d**) using the basic method.*

5.2 NMR

For NMR analysis, a Bruker Avance Ultrashield 400 spectrometer was used. Deuterated chloroform was used as a solvent. The chemical shifts are given in ppm; the coupling constants are given in Hz.

5.3.1. Substrate syntheses

The following substrates were synthesized via an isomerization reaction using $PdCl_2$. **4b** was synthesized via a demethylation reaction. The syntheses were performed following the published procedure.[4] The compounds were analyzed using NMR.

5.3.1.a

4-[(E)-1-Propenyl]-2-methoxyphenol (**1b**)

^{1}H NMR (400 MHz, Chloroform-d) δ 1.86 (dd, J = 6.6, 1.7 Hz, 3H, CH_3), 3.90 (s, 3H, OCH_3), 5.53 (s, 1H, OH), 6.08 (dq, J = 15.7, 6.6 Hz, 1H, H8), 6.32 (dq, J = 15.7, 1.7 Hz, 1H, H7), 6.79 – 6.92 (m, 3H, H arom).

The spectrum is in accordance with literature.[4]

5.3.1.b

p-[(*E*)-1-Propenyl]phenol (**4b**)
^{1}H NMR (400 MHz, Chloroform-*d*) δ 1.85 (dd, *J* = 6.6, 1.7 Hz, 3H, CH_3), 4.64 (s, 1H, OH), 6.08 (dq, *J* = 15.7, 6.6 Hz, 1H, CH), 6.33 (dq, *J* = 15.7, 1.8 Hz, 1H, CH), 6.76 (d, *J* = 8.6 Hz, 2H, H arom), 7.21 (d, *J* = 8.5 Hz, 2H, H arom).

The spectrum is in accordance with literature.[5]

5.3.1.c

2-[(*E*)-1-Propenyl]-6-methoxyphenol (**6b**)
^{1}H NMR (400 MHz, Chloroform-*d*) δ 1.91 (dd, *J* = 6.6, 1.7 Hz, 3H, CH_3), 3.89 (s, 3H, CH_3), 5.82 (s, 1H, OH), 6.29 (dq, *J* = 15.8, 6.6 Hz, 1H, H8), 6.60 – 6.74 (m, 2H, H arom,H7), 6.78 (td, *J* = 7.9, 0.5 Hz, 1H, H arom), 6.99 (dd, *J* = 7.9, 1.5 Hz, 1H, H arom).

The spectrum is in accordance with literature.[6]

5.3.1.d

4-[(*E*)-1-Propenyl]-2-methoxyphenyl acetate (**9b**)

^{1}H NMR (400 MHz, Chloroform-*d*) δ 1.88 (dd, *J* = 6.5, 1.7 Hz, 3H, CH_3), 2.30 (s, 3H, CH_3), 3.84 (s, 3H, OCH_3), 5.92 – 6.27 (m, 1H, H8), 6.37 (dq, *J* = 15.6, 1.7 Hz, 1H, H7), 6.66 – 7.15 (m, 3H, H arom).

The spectrum is in accordance with literature.[4]

5.3.1.e

4-[(*E*)-1-Propenyl]-1,2-dimethoxybenzene (**10b**)
^{1}H NMR (400 MHz, Chloroform-*d*) δ 1.87 (dd, *J* = 6.6, 1.7 Hz, 3H, CH_3), 3.87 (s, 3H, OCH_3), 3.89 (s, 3H, OCH_3), 6.10 (dq, *J* = 15.7, 6.6 Hz, 1H, H8), 6.34 (dq, *J* = 15.7, 1.7 Hz, 1H, H7), 6.52 – 7.12 (m, 3H, H arom).

The spectrum is in accordance with literature.[4]

5.3.1.f

5-[(*E*)-Prop-1-enyl]benzo[d][1,3]dioxolIsosafrole (**11b**)

^{1}H NMR (400 MHz, Chloroform-*d*) δ 1.85 (dd, *J* = 6.6, 1.7 Hz, 3H, CH_3), 5.93 (s, 2H, H1), 6.06 (dq, *J* = 15.7, 6.6 Hz, 1H, H9), 6.22 – 6.51 (m, 1H, H8), 6.54 – 6.80 (m, 2H, H arom), 6.79 – 6.96 (m, 1H, H arom).

The spectrum is in accordance with literature.[4]

5.3.1.g
m-[(*E*)-1-Propenyl]toluene (**14b**)

^{1}H NMR (400 MHz, Chloroform-*d*) δ 1.88 (dd, *J* = 6.5, 1.6 Hz, 3H, H9), 2.34 (s, 3H, H10), 5.96 – 6.32 (m, 1H, H8), 6.31 – 6.52 (m, 1H, H7), 6.70 – 7.08 (m, 1H, H arom), 7.08 – 7.24 (m, 3H, H arom).

The spectrum is in accordance with literature.[4]

5.3.1.h
p-[(*E*)-1-Propenyl]toluene (**15b**)

^{1}H NMR (400 MHz, Chloroform-*d*) δ 1.87 (dd, *J* = 6.5, 1.7 Hz, 3H, H9), 2.32 (s, 3H, H10), 6.18 (dq, *J* = 15.7, 6.6 Hz, 1H, H8), 6.30 – 6.51 (m, 1H, H7), 6.96 – 7.12 (m, 2H, H arom), 7.19 – 7.26 (m, 2H, H arom).

The spectrum is in accordance with literature.[4]

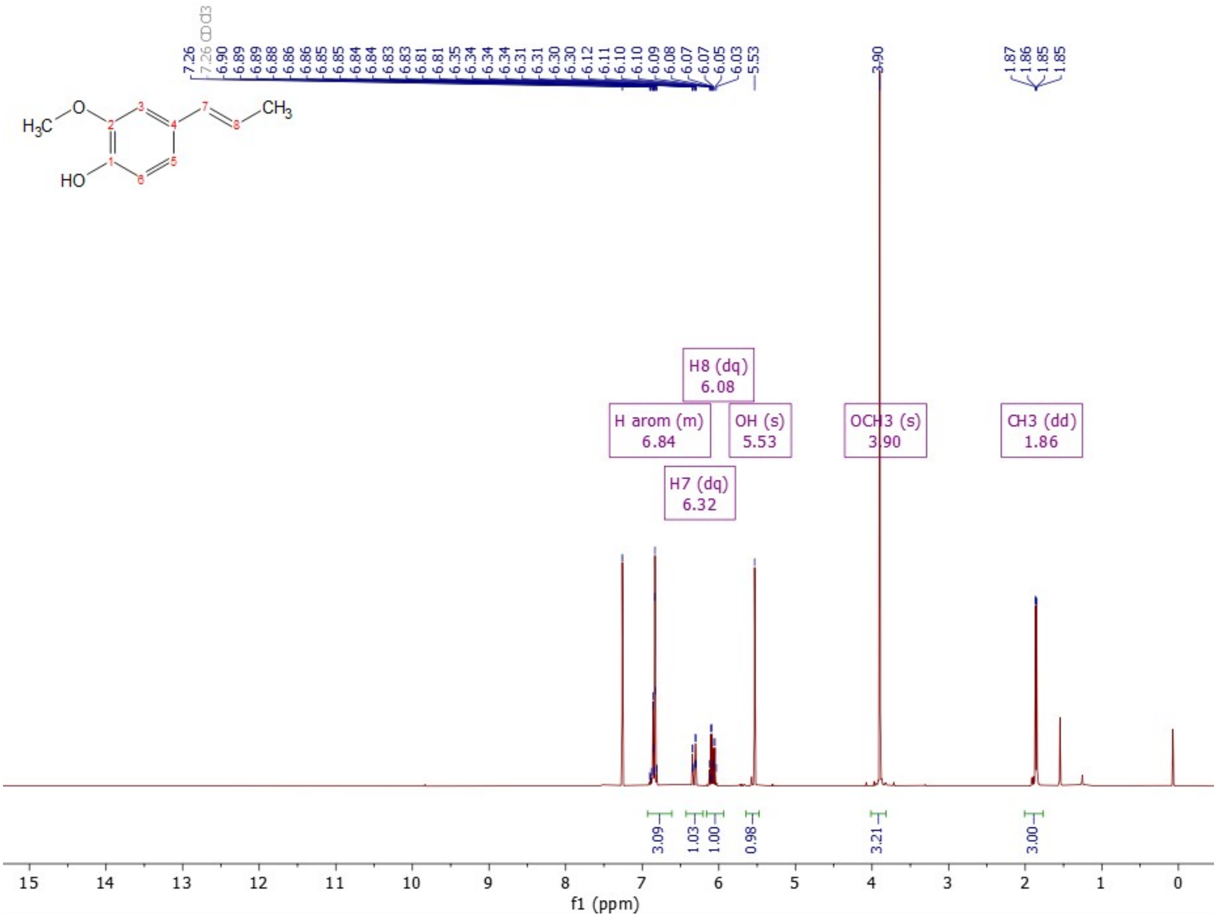

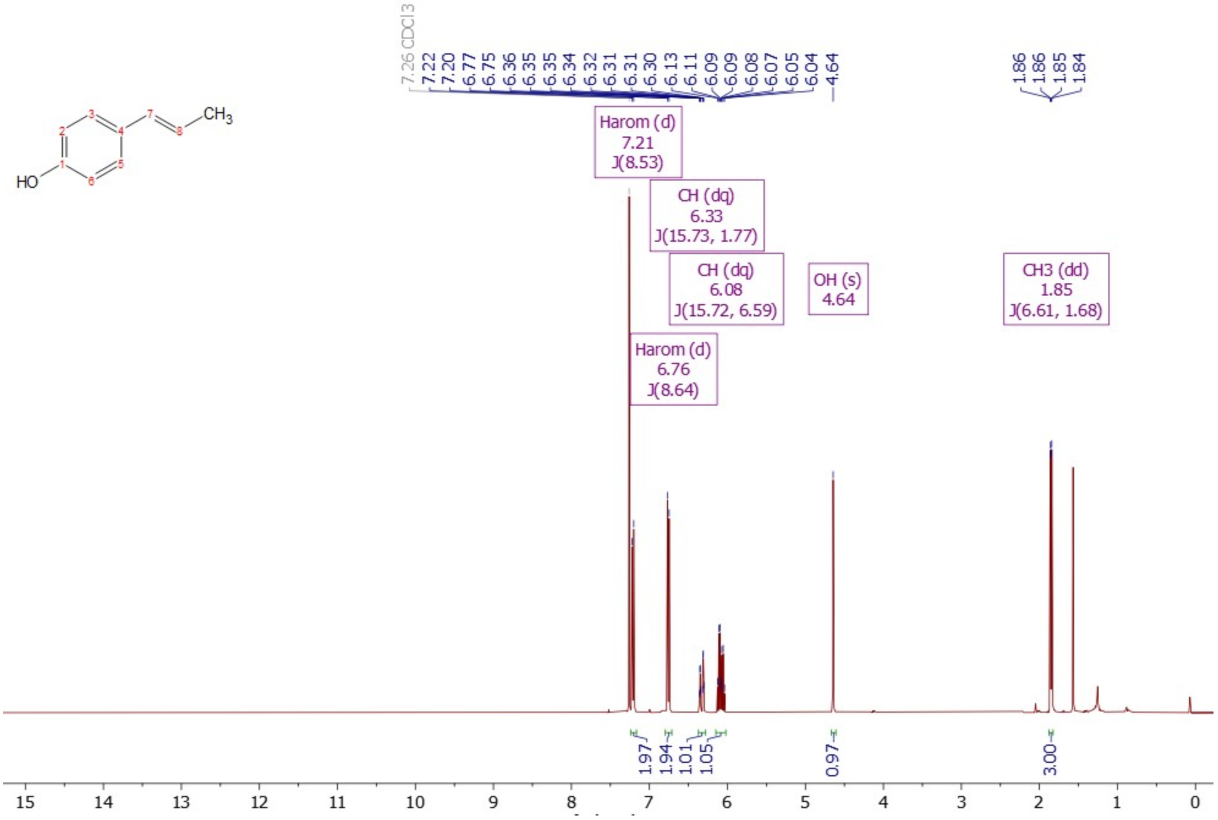
CH3
HO
7.26 CDCl3
Harom (d) 7.21 J(8.53)
CH (dq) 6.33 J(15.73, 1.77)
CH (dq) 6.08 J(15.72, 6.59)
OH (s) 4.64
CH3 (dd) 1.85 J(6.61, 1.68)
Harom (d) 6.76 J(8.64)
1.97
1.94
1.01
1.05
0.97
3.00

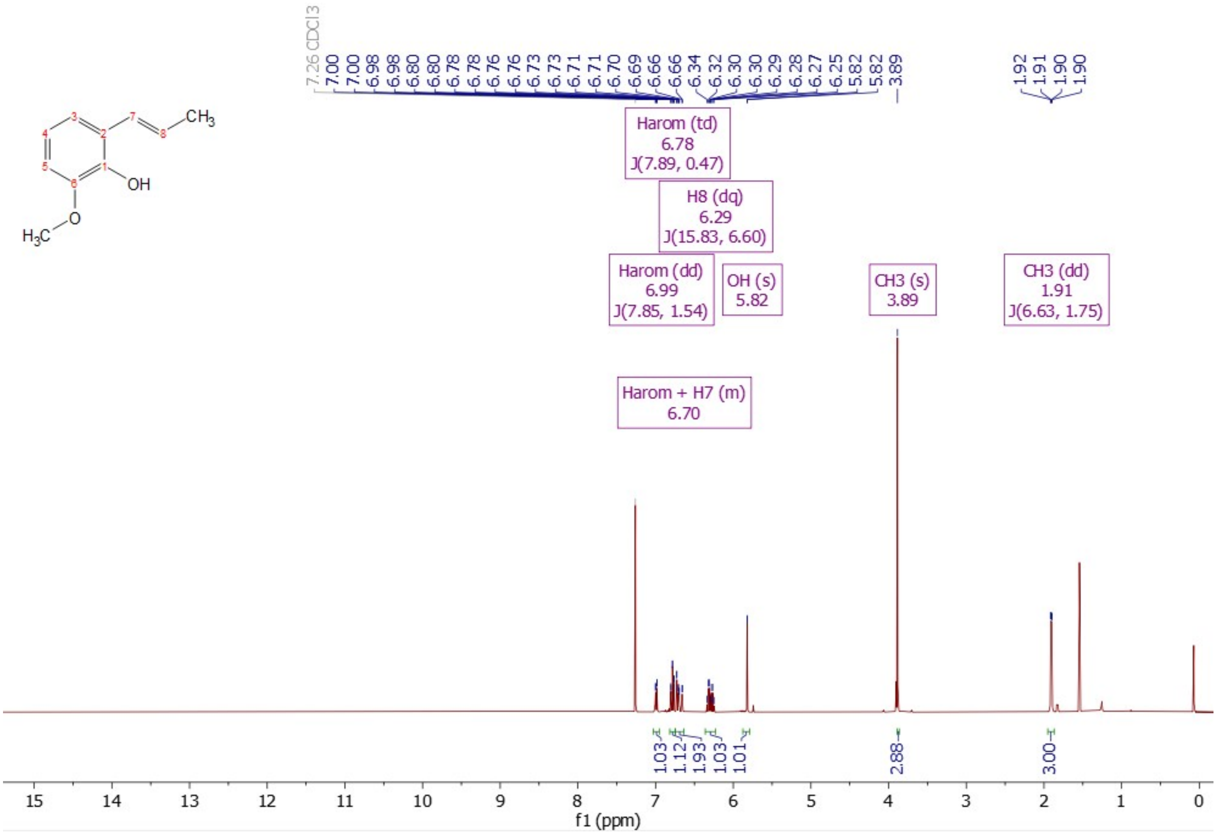
CH3
OH
H3C
O
7.26 CDCl3
Harom (td) 6.78 J(7.89, 0.47)
H8 (dq) 6.29 J(15.83, 6.60)
Harom (dd) 6.99 J(7.85, 1.54)
OH (s) 5.82
CH3 (s) 3.89
CH3 (dd) 1.91 J(6.63, 1.75)
Harom + H7 (m) 6.70
1.03
1.12
1.93
1.03
1.01
2.88
3.00
f1 (ppm)

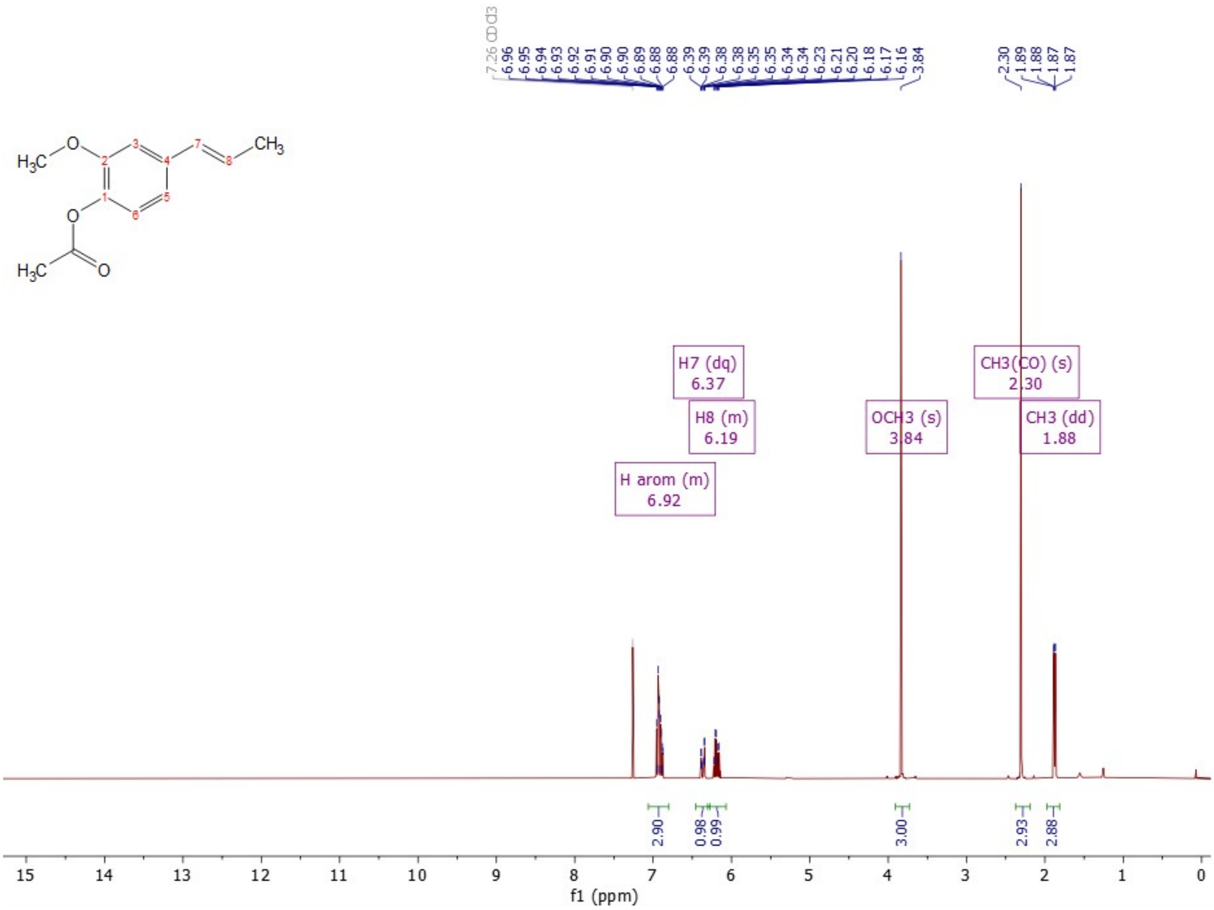
7.26 CDCl3
6.96 6.95 6.94 6.93 6.92 6.91 6.90 6.90 6.89 6.88 6.88 6.39 6.39 6.38 6.38 6.35 6.35 6.34 6.34 6.23 6.21 6.20 6.18 6.17 6.16 3.84
2.30 1.89 1.88 1.87 1.87
H7 (dq)
6.37
H8 (m)
6.19
H arom (m)
6.92
OCH3 (s)
3.84
CH3(CO) (s)
2.30
CH3 (dd)
1.88
2.90
0.98
0.99
3.00
2.93
2.88
f1 (ppm)

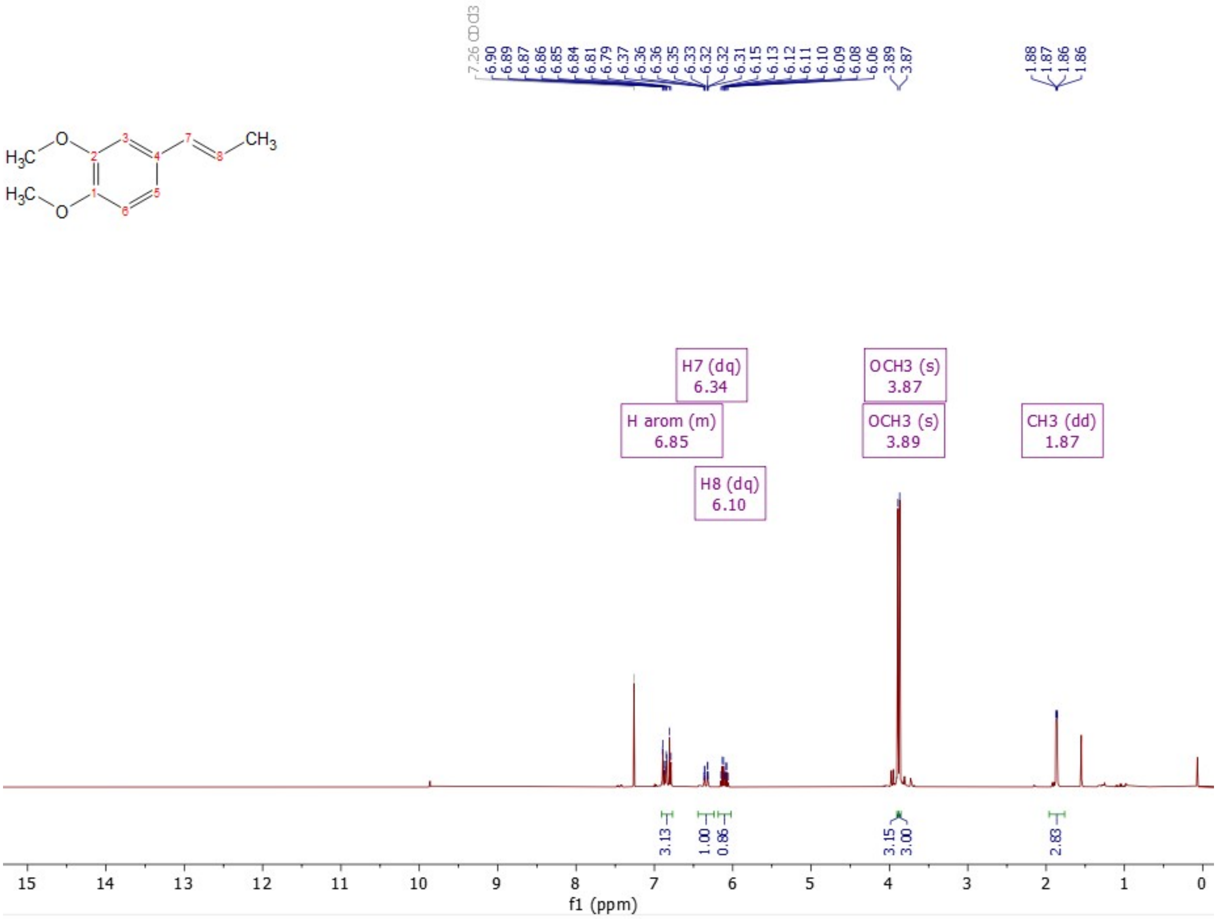
7.26 CDCl3
6.90 6.89 6.87 6.86 6.85 6.84 6.81 6.79 6.37 6.36 6.35 6.33 6.32 6.31 6.15 6.13 6.12 6.11 6.10 6.09 6.08 6.06 3.89 3.87
1.88 1.87 1.86 1.86
H7 (dq)
6.34
H arom (m)
6.85
H8 (dq)
6.10
OCH3 (s)
3.87
OCH3 (s)
3.89
CH3 (dd)
1.87
3.13
1.00
0.86
3.15
3.00
2.83
f1 (ppm)

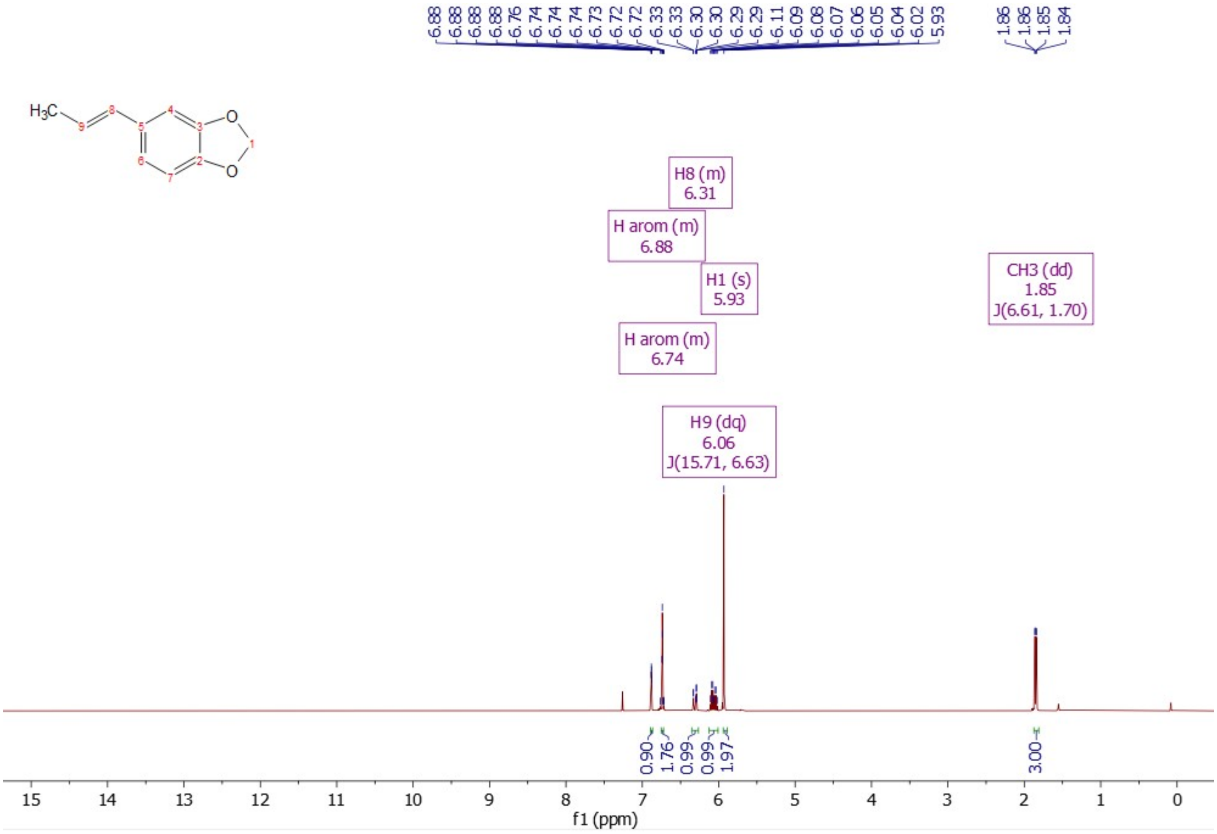
H8 (m)
6.31
H arom (m)
6.88
H1 (s)
5.93
CH3 (dd)
1.85
J(6.61, 1.70)
H arom (m)
6.74
H9 (dq)
6.06
J(15.71, 6.63)
f1 (ppm)

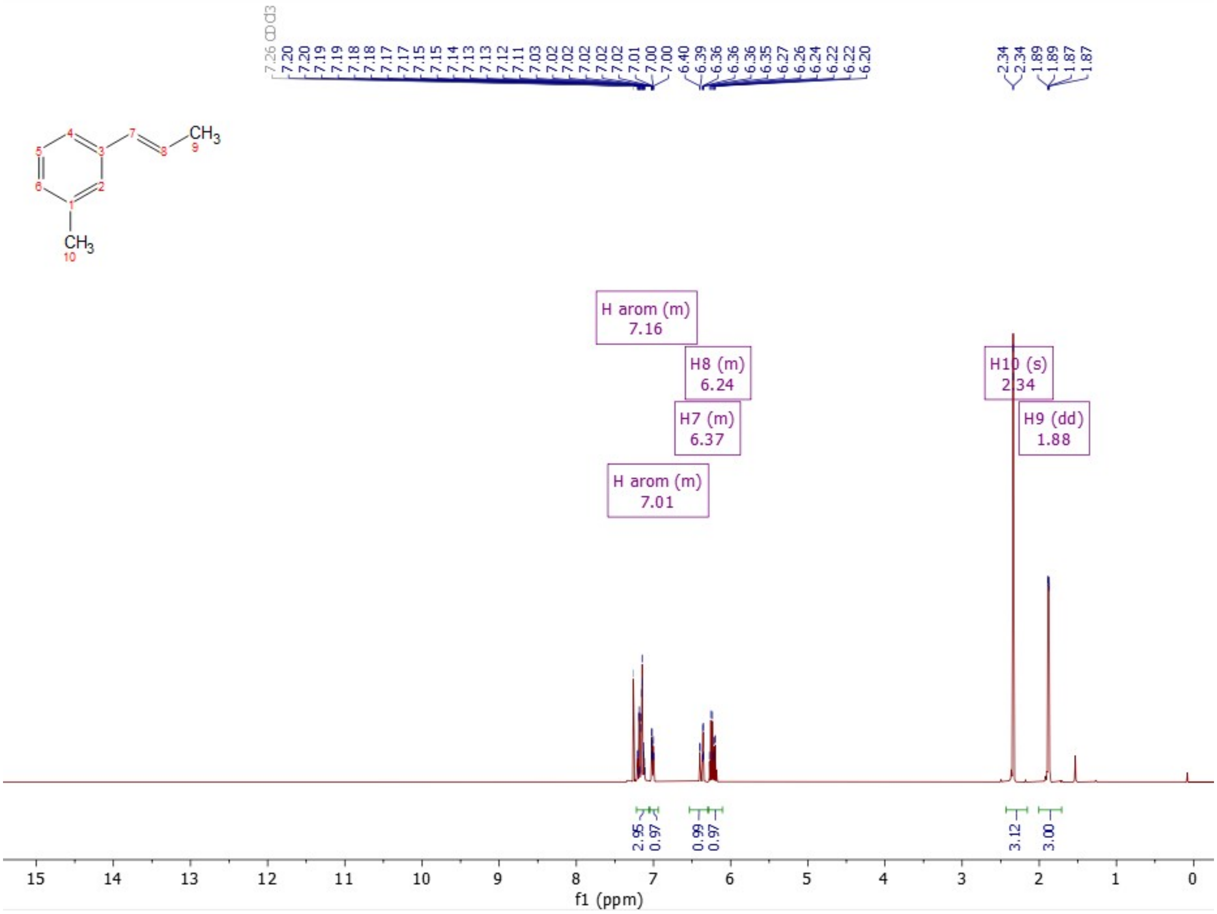
7.26 CDCl3
H arom (m)
7.16
H8 (m)
6.24
H10 (s)
2.34
H7 (m)
6.37
H9 (dd)
1.88
H arom (m)
7.01
f1 (ppm)

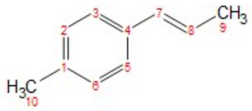

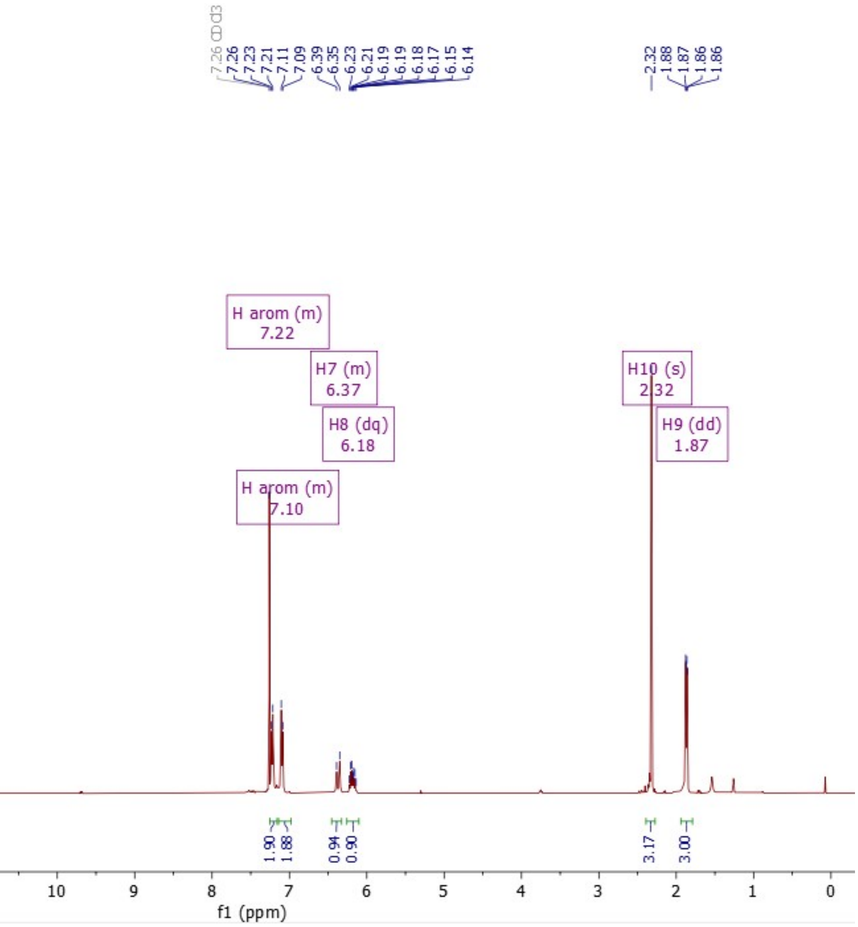

7.26 CDCl3
7.26
7.23
7.21
7.11
7.09
6.39
6.35
6.23
6.21
6.19
6.19
6.18
6.17
6.15
6.14
2.32
1.88
1.87
1.86
1.86
H arom (m)
7.22
H7 (m)
6.37
H8 (dq)
6.18
H arom (m)
7.10
H10 (s)
2.32
H9 (dd)
1.87
1.90
1.88
0.94
0.90
3.17
3.00
f1 (ppm)

5.3.2 *Preparative scale reaction*

NMR spectra of the product of the preparative scale using 50 mM isoeugenol were measured and confirmed that the obtained product was vanillin. The spectra are in accordance with literature[7].

^{1}H spectrum:

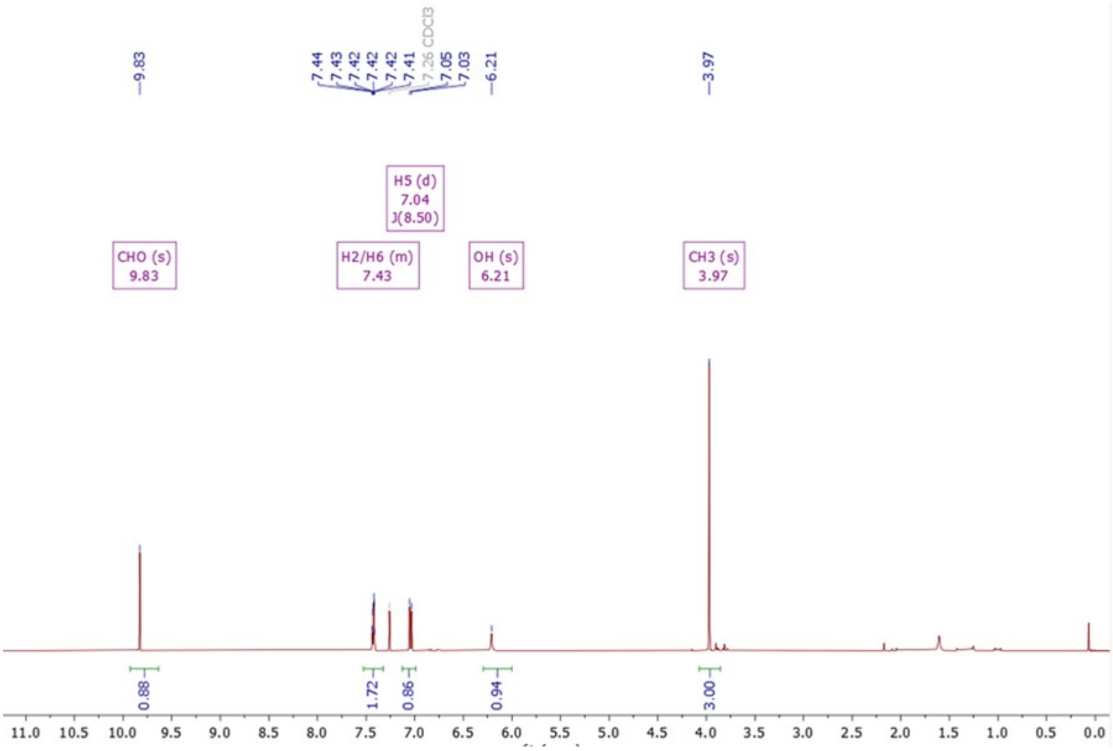

^{13}C-spectrum:

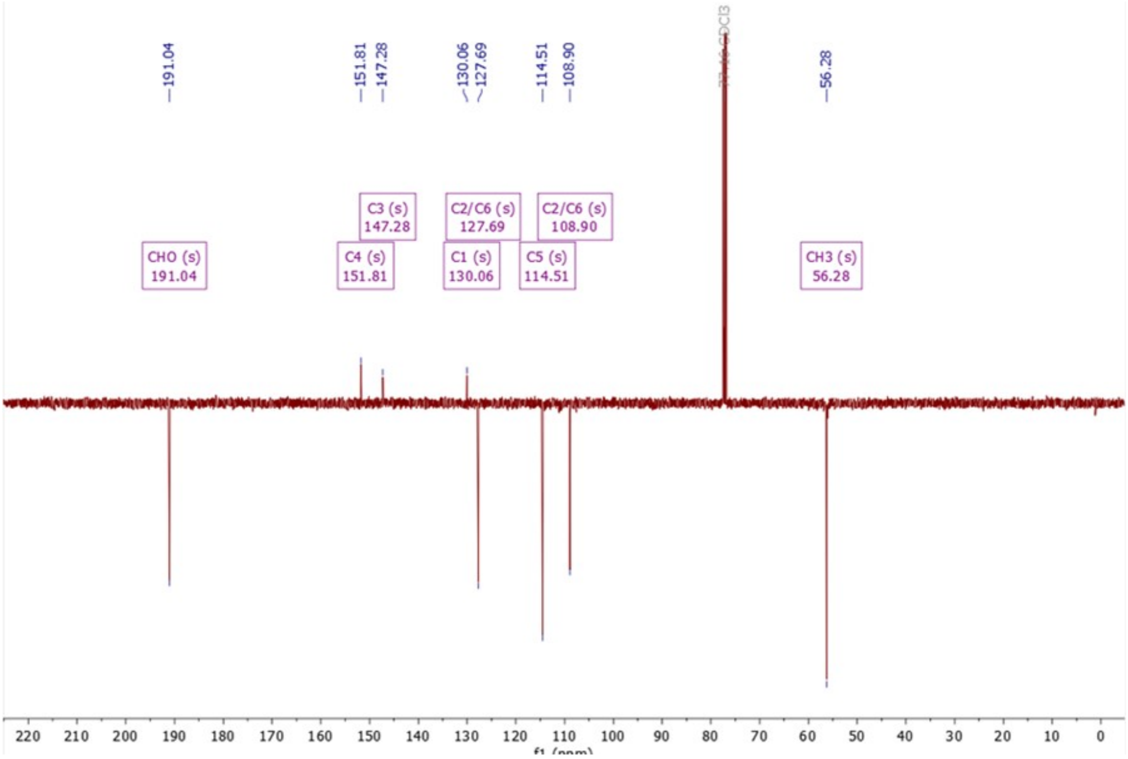

6. Green Metrics

The Green metrics' calculations were done based on the paper by Horvat and Iskra[8].

The following equations were used.

$$AE\ (\%) = \left(\frac{MWG\ product}{\sum MWGreactants * SC}\right) * 100$$

$$STY\ = \frac{mass\ of\ product}{reactor\ volume * time}$$

$$RME\ (\%) = \left(\frac{mass\ of\ product}{total\ input\ mass - mass\ recovered\ materials}\right) * 100$$

$$E - factor = \frac{total\ mass\ of\ waste}{mass\ product}$$

$$BY\ (\%) = \left(\frac{mass\ of\ product}{mass\ of\ catalyst}\right) * 100$$

AE *Atom economy*

STY *Space time yield*

RME *Reaction mass efficiency*

MW *Molecular weight*

SC *Stochiometric coefficient*

BY *biocatalyst yield*

The calculations were based on the data shown in Table 3 (entry 3), Figure 5, and on the data published by Lanfranchi *et al.* displayed in their Table 2 (entry 6)[9]. The results are shown in Table S. The amount of oxygen was estimated by adding up the estimated amount of dissolved oxygen (0.007 g/L)[10] and the assumption that oxygen makes up 20 % of the vial's headspace. The amount of NaOH to adjust the pH was not considered in the calculations. The E-factor was calculated without H_2O assuming that there is no need for special waste-water treatment.

Table S5: Comparison of the Green Metrics.

	Cascade with 5 mM eugenol		Cascade with 40 mM ferulic acid
	This paper	Lanfranchi *et al.* [9]	This paper
Yield (%)	81	91	92
BY (%)	15	4	23
AE (%)	67	67	78
STY ($gL^{-1}h^{-1}$)	0.002	0.013	0.029
RME (%)	0.06	0.07	0.53
E-Factor°	31	39	11

°Excluding water.

References

1 T. Saito, R. Aono, T. Furuya and K. Kino, *J. Biosci. Bioeng.*, 2020, **130**, 260–264.

2 L. Schober, J. Plewka, K. Sriwaiyaphram, B. Bielec, A. Schiefer, T. Wongnate, K. Magiera-Mularz, F. Rudroff and M. Winkler, *JACS Au,* 2025, DOI: 10.1021/jacsau.5c00456.

3 https://www.protocols.io/view/phosphate-buffered-saline-pbs-ewov1p8yvr24/v1 (accessed July 2025).

4 S. Giparakis, M. Winkler and F. Rudroff, *Green Chem.*, 2024, **26**, 1338–1344.

5 K. Nakayama, N. Maeta, G. Horiguchi, H. Kamiya and Y. Okada, *Org. Lett.*, 2019, **21**, 2246–2250.

6 H. Suga, Y. Hashimoto, Y. Toda, K. Fukushima, H. Esaki, A. Kikuchi, *Angew. Chem. Int. Ed.*, 2017, **56**, 11936–11939.

7 J. Jiang, J. Du, D. Liao, Z. Wang and Y. Ji, *Tetrahedron Lett.*, 2014, **55**, 1406-1411.

8 M. Horvat and J. Iskra, *Green Chem.*, 2022, **24**, 2073–2081.

9 E. Lanfranchi, V. Ferrario, S. Gandomkar, S. E. Payer, E. Zukic, H. Rudalija, A. Musi, I. Gaberscek, Y. Orel, D. Schachtschabel, C. Willrodt, M. Breuer and W. Kroutil, *ChemSusChem*, 2025, **18**, e202500387.

10 H. Patel and R. T. Vashi, in *Characterization and Treatment of Textile Wastewater*, Elsevier, Waltham, MA, 2015, pp. 21–71.
